# Protist diversity and community complexity in the rhizosphere of switchgrass are dynamic as plants develop

**DOI:** 10.1186/s40168-021-01042-9

**Published:** 2021-04-28

**Authors:** Javier A. Ceja-Navarro, Yuan Wang, Daliang Ning, Abelardo Arellano, Leila Ramanculova, Mengting Maggie Yuan, Alyssa Byer, Kelly D. Craven, Malay C. Saha, Eoin L. Brodie, Jennifer Pett-Ridge, Mary K. Firestone

**Affiliations:** 1 Bioengineering and Biomedical Sciences Department, Biological Systems and Engineering Division, Lawrence Berkeley National Laboratory, Berkeley, CA USA; 2 Institute for Biodiversity Science and Sustainability, California Academy of Sciences, San Francisco, CA USA; 3 Noble Research Institute, LLC, Ardmore, OK USA; 4 Institute for Environmental Genomics and Department of Microbiology and Plant Biology, University of Oklahoma, Norman, OK USA; 5 State Key Joint Laboratory of Environment Simulation and Pollution Control, School of Environment, Tsinghua University, Beijing, China; 6 Department of Environmental Science, Policy and Management, University of California, Berkeley, CA USA; 7 Ecology Department, Earth and Environmental Sciences, Lawrence Berkeley National Laboratory, Berkeley, CA USA; 8 Physical and Life Sciences Directorate, Lawrence Livermore National Laboratory, Livermore, CA USA

**Keywords:** Soil protist, Soil microbiome, Switchgrass, Rhizosphere, Community assembly

## Abstract

**Background:**

Despite their widespread distribution and ecological importance, protists remain one of the least understood components of the soil and rhizosphere microbiome. Knowledge of the roles that protists play in stimulating organic matter decomposition and shaping microbiome dynamics continues to grow, but there remains a need to understand the extent to which biological and environmental factors mediate protist community assembly and dynamics. We hypothesize that protists communities are filtered by the influence of plants on their rhizosphere biological and physicochemical environment, resulting in patterns of protist diversity and composition that mirror previously observed diversity and successional dynamics in rhizosphere bacterial communities.

**Results:**

We analyzed protist communities associated with the rhizosphere and bulk soil of switchgrass (SG) plants (*Panicum virgatum*) at different phenological stages, grown in two marginal soils as part of a large-scale field experiment. Our results reveal that the diversity of protists is lower in rhizosphere than bulk soils, and that temporal variations depend on soil properties but are less pronounced in rhizosphere soil. Patterns of significantly prevalent protists groups in the rhizosphere suggest that most protists play varied ecological roles across plant growth stages and that some plant pathogenic protists and protists with omnivorous diets reoccur over time in the rhizosphere. We found that protist co-occurrence network dynamics are more complex in the rhizosphere compared to bulk soil. A phylogenetic bin-based null model analysis showed that protists’ community assembly in our study sites is mainly controlled by homogenous selection and dispersal limitation, with stronger selection in rhizosphere than bulk soil as SG grew and senesced.

**Conclusions:**

We demonstrate that environmental filtering is a dominant determinant of overall protist community properties and that at the rhizosphere level, plant control on the physical and biological environment is a critical driver of protist community composition and dynamics. Since protists are key contributors to plant nutrient availability and bacterial community composition and abundance, mapping and understanding their patterns in rhizosphere soil is foundational to understanding the ecology of the root-microbe-soil system.

Video Abstract

**Supplementary Information:**

The online version contains supplementary material available at 10.1186/s40168-021-01042-9.

## Background

Plants evolved in a world dominated by prokaryotic and eukaryotic microbes [1], and through evolutionary time have established a dialogue with various soil microbial dwellers that are part of the rhizosphere microbiome [2]. One way plants influence the types of microorganisms that become part of their root microbiome is by releasing specific chemical cues via root exudates [3, 4]. Mesocosm-scale studies demonstrate that the chemical profile of root exudates changes as plants develop [4, 5] and these changes result in a “rhizosphere effect” that can be broadly defined as the influence of plant physiology on the physicochemical and biological properties of the root zone [6, 7]. This rhizosphere effect results in the modification of the abundance, diversity and composition of bacterial communities, and is frequently characterized by reduced diversity and more complex co-occurrence networks in the rhizosphere compared to bulk soil [5, 7, 8]. This effect also has broad ramifications for plant health, since members of the rhizosphere microbiome play a crucial role in important plant processes such as nitrogen (N) fixation, phosphorous (P) solubilization, production of plant growth regulators, and disease protection [9–12].

Although bacteria and fungi represent the most well-studied groups of microorganisms in the rhizosphere, they are only two components of the plant’s microbiome, which also includes viruses and protists that may also be influenced by the rhizosphere effect. The main groups of protists relevant to soil ecology—based on abundance and functional diversity—include the *Amoebozoa*, *Cercozoa*, *Ciliophora*, *Apicomplexa*, and *Chrysophiceae* [13–15]. Most soils are dominated by protistan consumers which span a wide range of feeding strategies, with prey including bacteria, fungi, algae, other microeukaryotes, and small protozoa [15]. Parasitism is another key protist functional role, particularly in groups such as the *Apicomplexa*, *Oomycota*, and *Ichtyosporea* [16], and phototrophy/mixotrophy in protists such as microalgae from the groups *Chrysophyceae* and *Chlorophyta* [17, 18].

Given their abundance and variety of functional trophic roles, protists play a critical part in shaping bacterial dynamics [19–21] and the abundances of other soil organisms [15]. Through their predatory activity, protists release nutrients from their prey’s biomass, making them available to plants and other organisms in their environment [22–25], while stimulating the rate of soil organic matter decomposition [9]. Although much attention has been directed toward the role of protists in N cycling, recent work has emphasized their other roles in soil ecology, including contributions to soil P mineralization [26]. For example, it has been demonstrated that protists interact with fungi by providing them with bacteria-derived nitrogen, which in turn improves plant nutrition by enhancing plant access to P and N [27, 28]. Furthermore, it has been shown that the selective pressure exerted by predatory activity of protists on soil microbes is also associated with bacterial populations with lowered susceptibility to infection by lytic phages [29, 30], indicating protists’ important roles as drivers of bacterial community assembly and evolution [31].

Despite their widespread distribution and ecological importance in soil microbial communities, protists remain a poorly understood component of the soil and rhizosphere microbiome [32]. This has begun to change as new molecular markers [33] and databases [34, 35] have been developed and improved. Several studies have deployed metabarcoding [36] and metatranscriptomics [37] in diverse soil types and revealed that soil protist diversity is higher than previously thought. Other molecular-based studies have shown that protist communities respond to environmental factors (including seasonal variations, water availability, and edaphic properties [15, 38]), external inputs such as fertilizers [39], vary with bacterial and fungal communities depending on the plant host type [40], and that consumer protists vary in the rhizosphere between healthy and diseased plants during plant establishment [41].

In a large-scale field experiment, we analyzed protist communities associated with the rhizosphere and bulk soil of switchgrass (SG) plants (*Panicum virgatum*) through multiple phenological stages, from early vegetative growth to senescence. The SG plants were grown at two marginal soil field sites where a limited reservoir of nutrients is available for plants [42]. We postulate that in this system, the rhizosphere effect influences community composition and network dynamics of rhizosphere protist communities. We used amplicon sequencing of ribosomal markers to assess the diversity and differential abundance dynamics of protists throughout SG growth stages in bulk and rhizosphere soil samples. Co-occurrence network analyses were used to analyze protist community dynamics as the SG plants grew. Our results reveal that the diversity of protists is lower in the rhizosphere than in bulk soil, indicating that the influence of plants on their microbiome cascades beyond bacterial communities and includes protist populations. We also demonstrate that protists network dynamics are more complex in the rhizosphere than in bulk soil (a phenomenon previously observed for bacterial communities), and that protist community assembly is controlled by dispersal limitation and homogenous selection. Overall, our results highlight the connection between plant and protist communities in a large-scale field experiment and illuminate the critical need to include protists in rhizosphere microbiome and functional studies.

## Results

### Protist diversity and community composition are influenced by soil properties

Protist communities were characterized in bulk and rhizosphere soil of switchgrass plants growing at two agricultural field sites with marginal soils. Soils were sampled at five times corresponding to different plant growth stages, including: early (T1) and late vegetative growth (T2), reproductive (T3), maximal growth (T4), and senescence (T5). Both field soils, a sandy loam (SL) and a clay loam (CL) are characterized as marginal based on their low total organic matter content and low plant available N and P (See Supplementary Dataset 1).

**Fig. 1:**
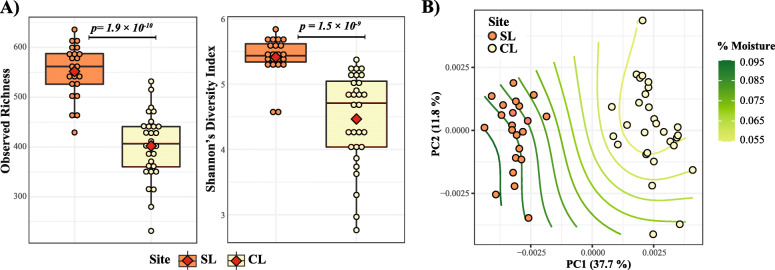
Diversity and community composition of protists communities in two marginal soil sites in southern Oklahoma. **a** Alpha diversity of protist communities in the two marginal soil sites. In each boxplot, a point represents a replicated sample per site and its calculated diversity index, and the diamond symbols represent the mean. The box boundaries represent the first and third quartiles of the distribution and the median is represented as the horizontal line inside each box. Boxplots whiskers span 1.5 times the interquartile range of the distribution. **b** Ordination plot depicting community structure of protist communities in the CL and SL sites calculated from a weighted Unifrac similarity matrix. The trend surface of the variable moisture was plotted onto the ordination space using the *ordisurf* function of the vegan package. CL = Clay Loam, SL = Sandy Loam; *n* = 30 for SL, *n* = 29 for CL

A total of 582 libraries were prepared for both sites, 293 from the CL site, and 289 from the SL site (detailed information can be found in the Supplementary Dataset 1, Tab 4). Together, the libraries yielded 6,533,093 18S rRNA sequences that were used to generate exact sequence variants (9304 exact sequence variants or ZOTUs, after dereplication). Non-protist groups were removed after taxonomic assignment, resulting in 4955 protist ZOTUs. Groups identified in both the SL and CL sites included protists from the divisions *Alveolata*, *Rhizaria*, *Amoebozoa*, *Stramenopiles*, *Excavata*, *Hacrobia*, *Archaeplastida*, *Apusozoa*, and *Proteoalveolata*. Diversity accumulation (rarefaction) curves showed that the detected communities were appropriately sampled for the CL and SL sites across sampling times (Supplementary Fig. 1). At the beginning of the experiment, bulk soil protist communities were significantly more diverse in the SL site relative to the CL site (alpha diversity, Observed richness and Shannon index, Wilcoxon test *p* < 0.001; Fig. 1a). Protist community composition was strongly affected by site (PERMANOVA: *df* = 1, *F* = 26.9, *p* = 9.9 × 10^−5^), which explained 35.5% of the data variation. An analysis of association between the ordination patterns of the protist communities and pH/moisture values further indicated significant associations between community structure and soil properties (*envfit* for 1^st^ and 2^nd^ axes, *p* = 0.06 for moisture and 0.007 for pH). Figure 1b shows the moisture values from each soil sample fitted onto the ordination biplot of protist communities, using a gam model to illustrate the observed relation between moisture and the observed patterns of community clustering (see Supplementary Fig. 2 for corresponding pH plot). Together, these results demonstrate that these two soils contain markedly different protist populations, and that protist community composition was influenced by the physicochemical properties of the soil.

### The diversity of protists is higher in bulk soil than in the rhizosphere

**Fig. 2:**
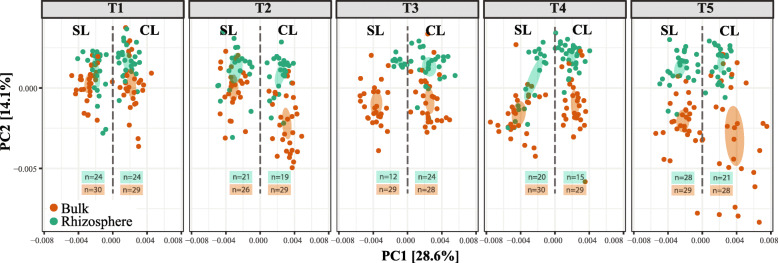
Community composition dynamics from sampling time T1 to T5 for protists in bulk and rhizosphere soil samples. The percent value for each axis represents the proportion of total variation explained. The ellipses were calculated around barycenters with a confidence level of 0.99 using the *stat_conf_ellipse* function in ggpubr v.0.2.4. SL = Sandy Loam site, CL = Clay Loam site. *N* values  correspond to the number of biological replicates that were used for analyses

At the initial stage of SG vegetative growth, protist community diversity (measured as observed richness and Shannon index) was not significantly different between bulk and rhizosphere soil, with the exception of the observed richness at the SL site. However, for all the subsequent sampling points, rhizosphere diversity was significantly lower than in paired bulk soils for both metrics (Supplementary Fig. 3), reaching its lowest levels during the reproductive growth stage (T3, Wilcoxon test *p =* 2.2 × 10^−7^ and 0.0006) of SG plants in the SL site, and during the maximal growth of the plants in the CL site (T4, Wilcoxon test *p* = 0.006 and 0.09). Sampling site (PERMANOVA: *df* = 1, *F* = 210.2, *p* = 9.9 × 10^−5^), time point (PERMANOVA: *df* = 4, *F* = 10.6, *p* = 9.9 × 10^−5^), and sample type (PERMANOVA: *df* = 1, *F* = 46.8, *p* = 9.9 × 10^−5^) all had significant effects on community composition, and explained 23.4, 4.6, and 5.1% of the observed data variation, respectively. An ordination plot comparing protist communities in the rhizosphere and bulk soil (Fig. 2) demonstrates that at both sites the rhizosphere community composition initially overlapped with the bulk protist community (T1), after which they diverged with time and continued plant growth (T3–T5). Soil moisture (*envfit* for 1^st^ and 2^nd^ axes, *p* = 0.012–0.001) and pH (*envfit* for 1^st^ and 2^nd^ axes, *p* = 0.001) had a significant effect on protist community composition in bulk and rhizosphere soil at both sampling sites and across sampling times (Supplementary Figure 4 and 5).

Because the same plants from our large-scale field experiment were sampled for both rhizosphere and bulk soil over 5 sampling points (T1 to T5), we were able to calculate the temporal variability of within sample alpha and beta-diversity for protist communities (Supplementary figure 6 and 7). Overall, the temporal variation of protist diversity was higher in the CL site than in the SL site (in both rhizosphere and bulk soil). These differences in temporal variability were significant for bulk communities between the two sites (Wilcoxon test *p* = 0.0003 and 4.86 × 10^−5^ for temporal variability in observed richness and Shannon index), but not within rhizosphere communities (Wilcoxon test *p =* 0.16 and 0.11). Similarly, the temporal variation in alpha diversity was significantly different between bulk and rhizosphere only in the CL site (Wilcoxon test *p* = 0.09 and 0.03 for temporal variability in observed richness and Shannon index). The significant high degree of temporal variability in alpha diversity of bulk protist communities was matched by high variability in community composition measured as beta-dispersion (Supplementary fig. 7). Comparison of β-dispersion between rhizosphere communities showed no significant differences between the two sites (Permutest *df* = 1, *F* = 0.76, *p* = 0.354), contrary to what was observed when comparing bulk communities (Permutest *df* = 1, *F* = 27.89, *p* = 0.001). Temporal dispersion was significant between bulk and rhizosphere from corresponding sites (SL, Permutest *df* = 1, *F* = 23.34, *p* = 0.001; CL, Permutest *df* = 1, *F* = 71.3, *p* = 0.001) as expected based on the beta-diversity analyses (Fig. 2).

**Fig. 3:**
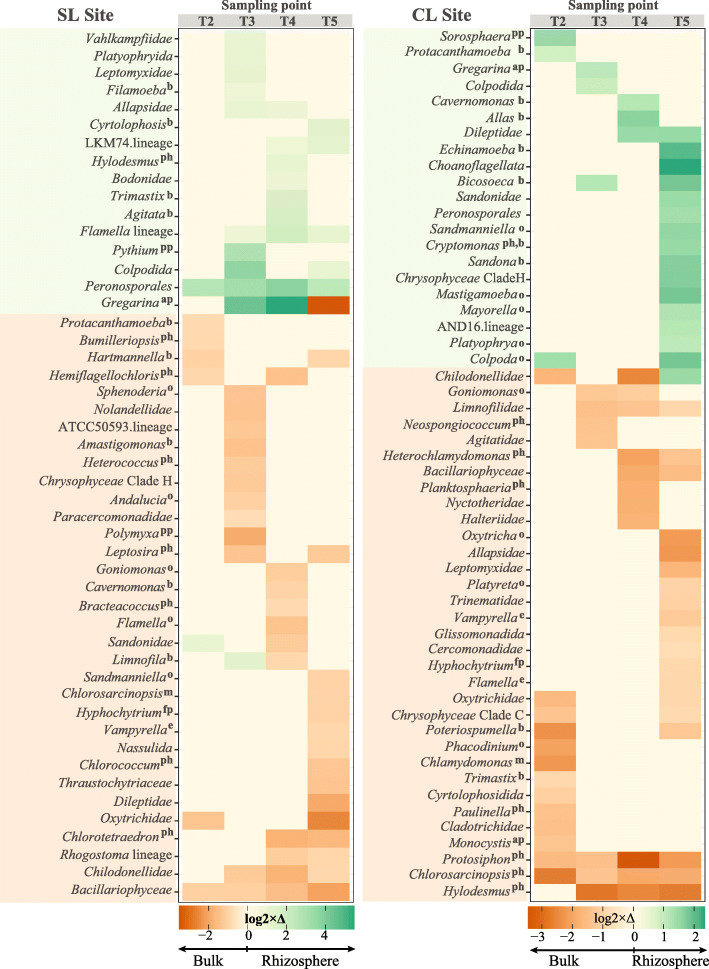
Differential abundance patterns of protist groups in rhizosphere and bulk soil over time. Green color indicates log2-fold abundance increase in the rhizosphere, while brown color indicates an increase of abundance in bulk soil. Only groups with a log2-fold change higher than 0.8 and lower than − 0.8 are represented in the figure. Significant differences for the groups had an FDR corrected *p* value < 0.01. Feeding/nutrition preferences are indicated based on published reports (see “Materials and methods” section) for those groups identified at the genus level and that were detected as prevalent in the rhizosphere. b = bacterivore, o= omnivore (feeds on bacteria and protists), e = eukaryvore, pp = plant pathogen, ph = photosynthesis, ap = animal pathogen, fp = pathogen of fungi. SL = Sandy Loam site, CL = Clay Loam site. For the SL site n-values were as follows: Rhizosphere-T2 = 21, T3 = 12, T4 = 20, T5=28; bulk-T2 = 26, T3 = 29, T4 = 30, T5 = 29. For the CL site n-values were: Rhizosphere-T2 = 19, T3 = 24, T4 = 15, T5 = 21; bulk-T2 = 29, T3 = 28, T4 = 29, T5 = 28

Differential abundances of detected protist groups were calculated for each time point to identify groups that became significantly more prevalent in the rhizosphere by comparing changes of each taxon across sampling times (Fig. 3). At the SL site, the order *Peronosporales* (which contains many plant pathogens) had become significantly more prevalent in the rhizosphere by T2 and remained so during all subsequent sampling times. Also at T2, the group *Sandonidae*, which includes bacterivore protists, was prevalent in the rhizosphere, and became more abundant in the bulk soil by T4. By the onset of reproductive growth (T3), we observed the emergence of protists from the genus *Gregarina* in the SG rhizosphere; this group includes pathogens of animals such as insects. These protists became even more prevalent during T4 in the rhizosphere, and then became more prevalent in bulk soil by T5. Also during T3, the bacterivores *Filamoeba* and *Limnofila* and protists from the families *Vahlkampfiidae*, and *Allapsidae* were significantly more prevalent in the rhizosphere, together with plant pathogenic protists from the genus *Pythium* (*Peronosporales*). Omnivorous groups, known to feed on bacteria and other protists, became prevalent in the rhizosphere by T3, including protists from the families *Flamellidae*, *Colpodida*, *Leptomyxidae*, and *Platyophryida*. The maximal growth stage of the SG plants at the SL site coincided with the emergence of bacterivore protists *Agitata*, the flagellate *Trimastix*, along with members of the family *Bodonidae*. By the point of SG senescence (T5), omnivore protists from the families *Colpodida* and *Flamellidae* were more abundant in the rhizosphere, together with the omnivore ciliate *Cyrtolophosis*.

At the CL site, we measured a similar transition in protist dynamics, with the largest number of rhizosphere-enhanced protist groups emerging by T5, the senescence phase. Putative plant parasitic protists from the genus *Sorosphaera* were significantly more prevalent during T2, together with the bacterivore *Protacanthamoeba*, and omnivore ciliates from the genus *Colpoda* (Fig. 3)*.* By T3, the rhizosphere of the SG experienced a significant increase in the abundance of bacterial predatory protists from the genus *Bicosoeca* and others from the family *Colpodida*, as well as the animal parasite *Gregarina.* During maximal growth (T4), groups that were significantly more prevalent in the rhizosphere included members of the family *Chilodonella* as well as bacterivores identified as the naked amoeboflagellate *Cavernomonas*, and the flagellate *Allas*. At the senescence (T5) stage of SG growth, we measured the emergence of many microbial predatory protists, including several amoebae belonging to the genera *Echinamoeba*, *Mastigamoeba*, *Mayorella*, and the amoebozoan lineage AND16. Flagellated protists such as *Sandona*, *Cryptomonas*, *Bicosoeca*, together with the ciliates *Sandmanniella* and *Platyophrya* (T5) and algae from the *Chrysophyceae* Clade H, were also more prevalent in the rhizosphere at plant senescence.

Changes in abundance of different protist groups were also detected in the bulk soil over time (Fig. 3). These changes in community prevalence are likely the result of seasonal changes in precipitation and soil temperature that occurred during the field sampling period (June to November, see supplementary dataset 1, tab 3). Green algae were among the more prevalent groups in the bulk soil at both sites across sampling times (Fig. 3). These groups included *Hemiflagellochloris* at T2; *Heterococcus*, *Leptosira*, and *Neospongiococcum* at T3; *Bracteacoccus* and *Planktosphaeria* at T4; and *Chlorococcum*, *Chlorotetraedron*, *Protosiphon*, *Chlorosarcinopsis*, and *Hylodesmus* at T5. The presence of these algae was accompanied by the emergence of the algae-feeding amoeba *Vampirella* during T5. Other groups detected in bulk soil included known omnivores such as *Sphenoderia* and *Andalucia* at T3; *Goniomonas* (T3/T4), *Flamella*, *Oxytricha*, and *Platyreta* at T4, and *Phacodinium* and *Sandmaniella* at T5*.*

### Co-occurrence network complexity of protists is higher in the rhizosphere

**Fig. 4:**
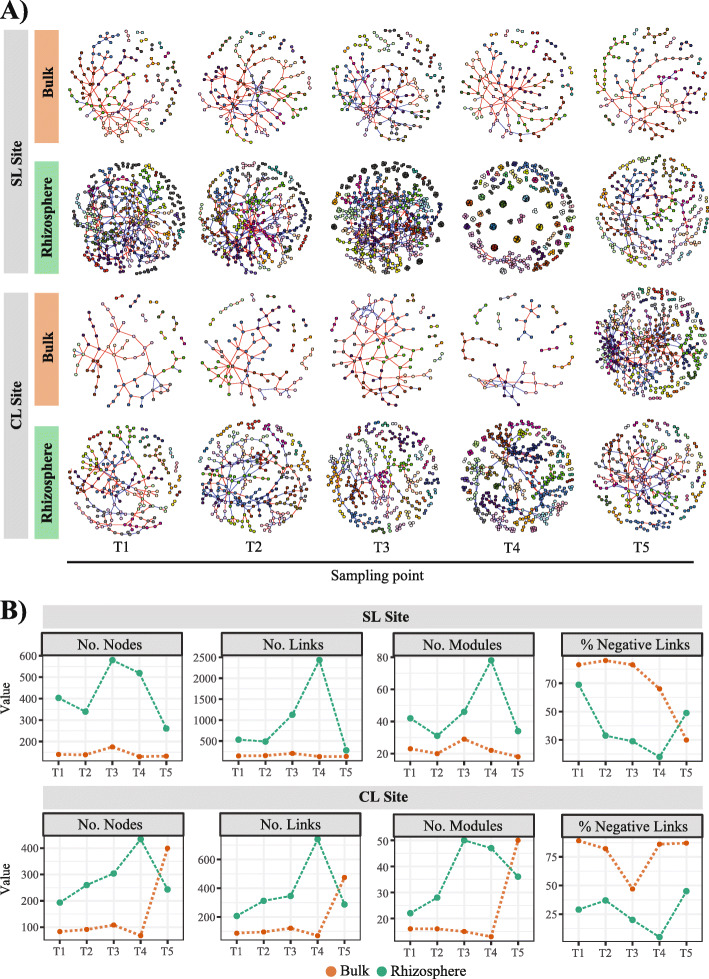
**a** Succession of rhizosphere and bulk soil networks for protist communities over time. The five sampling points corresponded with different developmental stages of switchgrass plants in two sampling sites. Networks represent RMT co-occurrence models from biological replicates (minimum of 10) at each sampling point, where nodes represent ZOTUs or exact sequence variants, and links between nodes represent significant correlations. Modules are randomly colored. Red and blue links represent significant negative and positive correlations. **b** Network topological parameters for both sites over time for bulk and rhizosphere protists networks. SL = sandy loam site, CL = clay loam site

We used random matrix theory-based network analysis to characterize changes in protist co-occurrence network assembly in the rhizosphere and bulk soil of SG. Networks of protists in rhizosphere communities consistently differed from those present in the bulk soil, during each sampling time and at both sampling sites (Fig. 4a). Protist community data reflect larger co-occurrence networks (more nodes) in the rhizosphere from both sites; these were more connected (larger number of links) and formed more modules than in the bulk soil (Fig. 4a, b). The topological parameters (number of nodes, links, modules) of the rhizosphere networks from both sites increased with time from T1 to T4, and then decreased to levels similar to those calculated for bulk networks at T5 (plant senescence). Conversely, network size, connectivity, and the number of modules in bulk soil remained constant across all time points, with the exception of T5 at the CL site, where these topological parameters increased and converged to levels similar to those of the rhizosphere network for the same time point. Another difference between the rhizosphere and bulk co-occurrence networks was the type of associations that dominated each environment (Fig. 4b). Bulk networks from both sites had a higher percent of co-exclusion associations (negative correlations) than those detected in the rhizosphere during all sampling points, with the exception of the SL site bulk network for T5.

We defined putative roles for protists that were part of the constructed networks by classifying network nodes based on their within-module connectivity (z-score) and participation coefficient (p-score) (Supplementary Table 1). Most nodes were identified as peripheral (99.1%, regardless of site or sampling time), and the remaining nodes were module hubs and connectors. Module hubs represent highly connected nodes within modules, and connectors are nodes that connect modules. Due to their contribution to network topology, module hubs and connectors have been proposed to represent potential keystone taxa [43]. Here, we refer to these network-relevant nodes as *hub taxa* that may represent important organisms contributing to the stability of overall protist communities.

Protist hub taxa changed over time at both sites, and relatively few groups persisted as hub taxa over time (Table 1). At the SL site, T1 contained hubs identified as the plant parasite *Pythium*, the bacterivore *Paracercomonas*, the omnivore *Rhogostoma*, and the eukaryvore *Bresslaua*. By T2, the hub taxa changed, and included protists from the family *Sandonidae*, the order *Cercomonadida*, and the class *Heterolobosea*. At T3, hub taxa was mainly represented by omnivore protists including *Acanthamoeba*, *Stenamoeba*, and *Cercomonas.* During the maximal growth of SG (T4) at SL site, only one hub was detected and identified as member of the order *Cercomonadida*. By the SG senescence stage (T5), hubs included the bacterivore *Thaumatomonas*, and protists from the family *Cercomonadidae* and the order *Cryomonadida*. Similar dynamism among hub groups was detected at the CL site. T1 hub taxa included the omnivore *Euglypha*, the algae *Parietochloris*, and other taxa identified at the level of family such as *Allapsidae* and *Trebouxiophyceae*. No hub taxa groups were identified during T2, and by T3, hub taxa groups included the plant parasite *Polymyxa*, the omnivore *Eocercomonas*. At T4, hub taxa included omnivore protists from the genera *Trinema* and *Acanthamoeba*. The omnivore *Trinema* continued to be a hub taxa at plant senescence (T5), together with the plant pathogen *Polymyxa* and the bacterivore *Paracercomonas*.

**Table 1:**
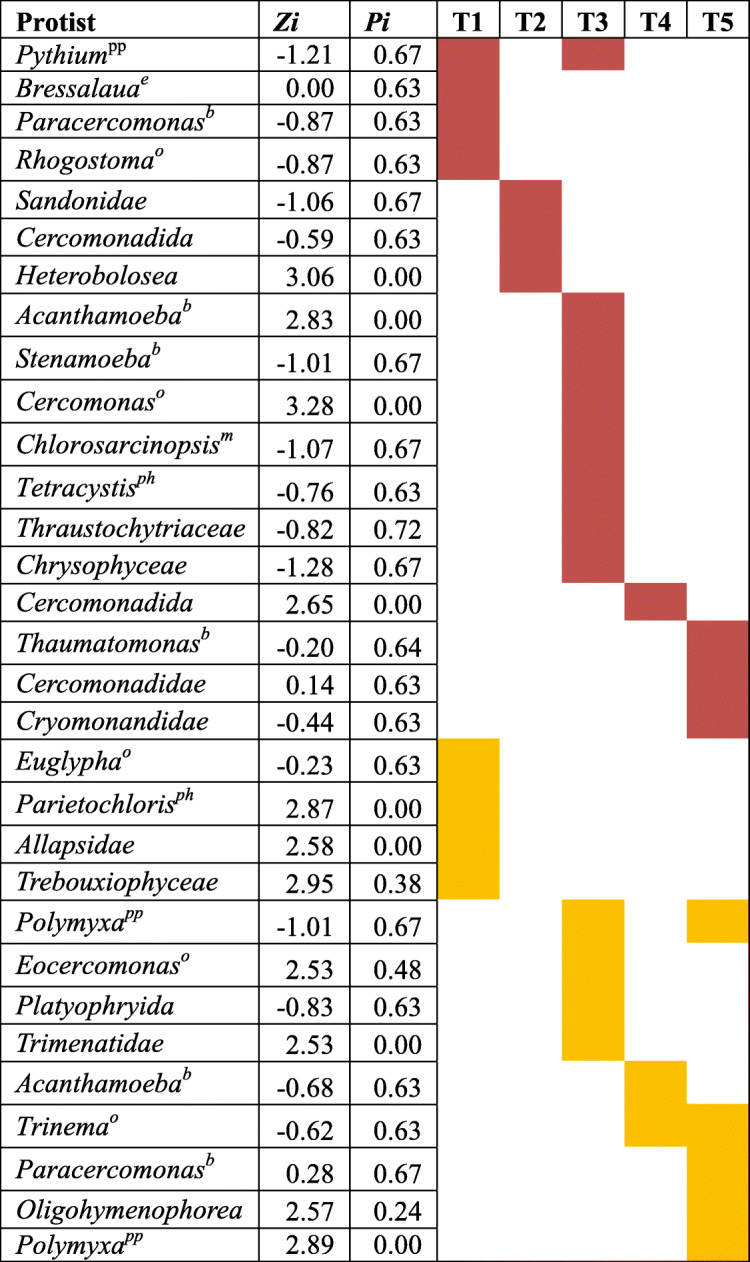
Protists identified as putative hub taxa within the rhizosphere networks from the Sandy Loam (red box) and Clay Loam (yellow box) sites

Hub taxa are module hubs and connectors which are identified based on their within-module connectivity (*Zi* > 2.5) and among-module connectivity (*Pi* > 0.62). Their removal from their corresponding networks may cause modules and networks to disassemble. Boxes filled in red color correspond to times in which a protist was identified as keystone element of a rhizosphere network from the Sandy Loam site while yellow filled box correspond to keystone groups at the Clay Loam site. T1 to T5 indicate the different sampling times. Feeding/nutrition preferences are indicated based on published reports (see materials and methods) for protists identified at the genus level. *b* bacterivore, *e* eukaryvore, *o* omnivore (feeds on bacteria and protists), *ph* photoautotroph, *m* mixotroph, *pp* plant pathogen

### Dispersal limitation and homogeneous selection shape rhizosphere protist communities

**Fig. 5:**
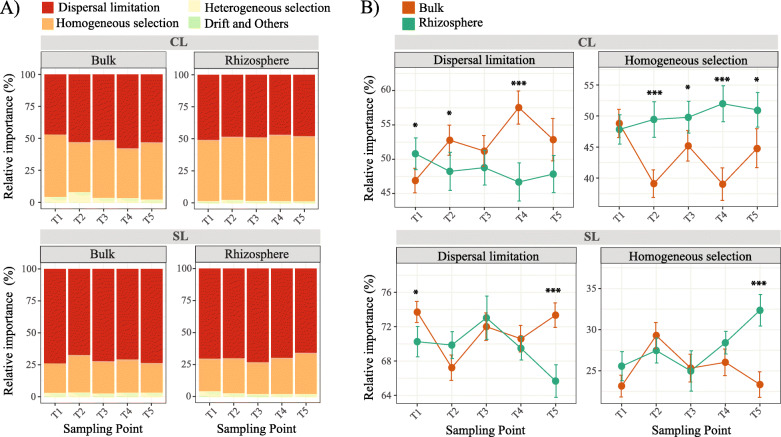
Relative importance of different ecological processes in protist community assembly. **a** Dispersal limitation and homogenous selection were the most influential ecological processes in both sampling sites for bulk soil and rhizosphere communities across sampling times. **b** Comparison between bulk soil and rhizosphere show that rhizosphere protists are under higher homogeneous selection but lower dispersal limitation during the growth and/or senescence of switchgrass. Significance is based on bootstrapping with 1000 replications. *P < 0.1; **P < 0.05; ***P < 0.01

We inferred community assembly mechanisms with a phylogenetic bin-based null model (iCAMP) [44] and found that dispersal limitation and homogenous selection were the key processes driving protist community assembly in both sampling sites during the five sampling times. Dispersal limitation had a larger effect on community assembly at the SL site (67–73%), followed by homogenous selection (23–29%) (Fig. 5a). At the CL site, both dispersal limitation and homogenous selection had similar effects on protist community assembly, 46–57% and 39–48% respectively (Fig. 5a). A comparison between bulk and rhizosphere at each sampling site showed that homogenous selection was more influential in the rhizosphere of the CL site from late vegetative growth through SG senescence (T2 to T5), and from maximal growth to senescence (T4 to T5) at the SL site (Fig. 5b). In contrast, the influence of dispersal limitation was overall higher in the bulk soil at the CL site, while remaining similar between bulk and rhizosphere from T2 to T4 at the SL site and then higher in bulk soil by T5 (Fig. 5b).

## Discussion

In this study, we explored protist communities, their dynamics, and mechanisms of assembly in rhizosphere and bulk soil of switchgrass plants grown in two marginal soil sites in southern Oklahoma. Because variations in 18S rRNA copy number per eukaryotic cell limits the quantitative value of 18S rRNA barcoding [45, 46], and given that 18S rRNA primers have the potential to amplify non-targeted groups, we decided to (1) remove non-targeted groups from our datasets prior to community analyses and (2) make a point of comparing the prevalence within a taxon across sampling times to reduce the effect of 18S rRNA copy number (as per Berdjeb et al. [46]). We found that the diversity and composition of protist communities are influenced by environmental properties (pH, water content, soil type) that commonly define important components of microsite niche heterogeneity in soil (Fig. 1). These results are in agreement with previous work reported by Fiore-Donno et al. [38], who demonstrated that Cercozoa protists were influenced by edaphic factors including water and clay content over the course of a growing season in a temperate grassland site. Together, this evidence suggests that protists are constrained by similar edaphic factor as their main prey, bacteria [47, 48].

The presence of plant roots is known to differentiate soil niche heterogeneity in rhizosphere soil, likely due to the release of rhizodeposits that modify pH of the surrounding soil, as well as the availability of C, water, and oxygen [49]. The influence of plant roots on soil properties is known to select for a subset of bulk soil bacterial populations with the genetic and metabolic traits to subsist and grow in the rhizosphere, which in turn results in the reduced microbial diversity that characterizes the rhizosphere effect [7, 50–52]. The rhizosphere protist community composition patterns we measured parallel those that have previously been observed for rhizosphere bacterial communities [5, 8] (Fig. 2). As SG plants grew, the diversity of protist communities in their rhizosphere declined and reached the lowest levels by the plant’s reproductive stage (T3) and maximal growth (T4), only to recover to diversity levels similar to the early vegetative stage during the plant’s maximal growth and senescence (T4 and T5) (Supplementary Fig. 3). This likely reflects the amount and composition of SG rhizosphere deposits, which undoubtedly follow different temporal patterns in this perennial grass than what has been previously observed in annuals. In addition to the reduction of rhizosphere protist diversity and dynamism with time, our study identifies select groups that became significantly more prevalent in the SG rhizosphere. With the exception of putative plant pathogens, very few of these groups (that included mostly bacterivore protists) continued to dominate in the rhizosphere across all plant growth stages (Fig. 3).

Phototrophic protists were significantly more prevalent in the bulk soil of our marginal soil sites across sampling times and were accompanied by algivorous protists (Fig. 3). While somewhat surprising, this may be due to our sampling depth (from 0 to 20 cm) and sampling strategy. After soil cores were extracted from the ground, roots were separated and washed to generate the rhizosphere samples. Then, the remaining soil was mixed and used as the bulk soil; this bulk soil portion likely included the first centimeters of the soil surface, which may have been colonized by a biocrust, particularly in early timepoints when there was relatively little plant cover. This could explain why photosynthetic protists were prevalent at several timepoints. It is also possible that the site’s initial soil cultivation may have resulted in the mixing of surface crust-associated photosynthetic protists throughout the top 20 cm of the soil profile.

Changes in protist diversity were accompanied by modifications in community composition over time (Fig. 2), and our analyses of temporal variability demonstrated that community variation for protist alpha and beta-diversity was larger at the clay loam (CL) site than in the sandy loam (SL) site (supplementary figures 6 and 7). These analyses also showed that the observed temporal variation for protist communities was larger in bulk soil than in the rhizosphere, and non-significant when compared between rhizosphere communities from the CL and SL site. Ecological theory predicts that more open habitats with large species pools, bulk soil in our case, should vary more through time [53], as we observed. Similarly, our temporal variation analyses show that the type of soil (CL vs. SL sites) plays a key role in community dynamics. In the rhizosphere, protist communities appear more stable (in terms of temporal variations of diversity and composition) than in bulk soil, possibly due to the steady supply of bacterial prey and to the more homogenous, although still dynamic [54], niches surrounding plant roots.

Rather than representing interactions, molecular ecological networks can be applied to evaluate the complexity of targeted communities [55, 56] and have been successfully used to analyze the effects of environmental properties on microbial communities [57]. Multiple studies indicate that the rhizosphere effect filters bacterial communities, influences their assemblages, and promotes more complex network dynamics in the rhizosphere than in bulk soil [8, 58, 59]. However, very few studies have examined analogous patterns for protists, particularly at the field scale and over time [38]. Based on previous reports analyzing patterns observed for bacteria, we postulate that the identified changes in protist diversity and prevalence at the rhizosphere level are accompanied by changes in protist network dynamics which may result from direct root-based environmental filtering of the bacterial food source and direct filtering of protist populations. To test this, we first measured changes in community composition between bulk soils for our sampling points and demonstrated that in both sites the rhizosphere’s protist community composition changed relative to the bulk soil over time (Fig. 2). Co-occurrence network analyses further demonstrated these changes in protist community assembly where protist rhizosphere assemblages formed larger and more complex co-occurrence networks than bulk soil communities that remained unchanged with the exception of T5 for the CL site (Fig. 4a, b). Our analyses also showed that rhizosphere networks developed in parallel with plant growth (from vegetative growth to senescence) (Fig. 4a). Network complexity (number of nodes, links, and modules) reached its maximum level during reproductive and maximal SG growth stages (T3 and T4), then decreased during senescence, ultimately reaching levels similar to those of the early vegetative stage (T1) (Fig. 4b). In contrast to the rhizosphere networks, nodes from bulk networks were predominantly connected through negative associations. Co-occurrence studies have demonstrated that rather than representing competition, negative correlations may be a result of abiotic variation (niche heterogeneity) in the environment of the analyzed communities [60, 61]. Here, we posit that the higher percentage of negative correlations observed in bulk soils is the result of greater soil heterogeneity, and a larger diversity of environmental niches. In comparison with bulk soil, the rhizosphere represents a more homogeneous environment that, although changing over time as a result of modifications in the plant’s physiology, selects for reduced protist diversity occupying more controlled niches near the plant root.

Module hubs and connectors are network nodes that upon their removal may cause modules or networks to disassemble [62, 63], and may represent keystone species in an ecosystem [64, 65]. Most of the hub protist taxa that we identified in the rhizosphere association networks were identified as hub taxa at only one sampling time (Table 1, Supplementary Fig. 2) suggesting that the roles played by these protists differed across plant growth phases. Several protist groups were exceptions to this episodic pattern with some organisms appearing as hub taxa at a site during two of the five growth phases. For example, two taxa, a *Pythium* and *Polymyxa*, were identified as hub taxa in the SL and CL sites during the time points T1 and T3, and T3 and T5, respectively. *Pythium* and *Polymyxa* genera include plant pathogenic organisms that may persist in the rhizosphere over time [66, 67]. Another group of recurring keystone protists was represented by taxa identified as members of the order Cercomonadida including *Cercomonas*, *Paracercomonas*, and *Eocercomonas*, which are able to survive on an omnivorous diet [68, 69]. Several other protists with the capacity to survive on an omnivorous diet were also detected as keystone taxa of the rhizosphere association networks; these organisms included naked and testate amoeba from the genera *Rhogostoma*, *Acanthamoeba*, *Euglypha*, and *Trinema* [70–72]*.*

As plants grow and develop, they release root exudates, including different metabolites that regulate the types of bacteria that become part of the rhizosphere microbiome during different developmental stages of the plant [4, 73]. Our results suggest that plants may also influence the types of protists that become part of their rhizosphere microbiome. In many perennial grasses, young plants allocate more C to the roots, whereas older plants allocate C to shoots, reducing the inputs of total C to the rhizosphere [74] and also changing exudate composition [4, 75]. The dynamic selection by the plant for its bacterial and fungal communities may also explain the continuous changes in prevalent protist groups and network hubs that we observed in the rhizosphere (Fig. 3, Supplementary Table 1). Protists are selective feeders that choose their prey based on chemical signals released by the potential prey as well as morphological characteristics such as size [76]. In this way, protists that are able to select and survive on more than one type of diet, such as omnivorous protists, may be better suited to occupy key roles in the different niches offered by the plant in its rhizosphere.

We assessed protist community assembly processes in both sampling sites to further understand the mechanisms governing the observed differences in protist community properties between bulk and rhizosphere over the phenological stages of SG. Dispersal limitation and homogenous selection were identified as the main processes of protist community assembly in the SL and CL sampling sites (Fig. 5a). Dispersal limitation refers to a mechanism in which the movement or establishment of individuals to a new location is restricted, leading to dissimilar structures among communities [77]. Homogeneous selection, on the other hand, is a mechanism in which homogenous abiotic and biotic environmental conditions lead to more similar structures among communities [77]. The influence of homogenous selection was higher in the rhizosphere as the plant developed (T2 to T5 at CL and T3–T5 at SL), possibly due to the plants’ control over the biotic and abiotic properties at the rhizosphere level and the formation of more homogenous, but dynamic, environmental conditions [54] (Fig. 5b). These results parallel our observation of a higher proportion of positive correlations among protists in the rhizosphere compared to bulk soil, as more positive correlations may result from lower niche heterogeneity [60, 61]. Dispersal limitation was higher in bulk soil than in rhizosphere, and its differential influence was more prominent at the CL site (Fig. 5b). This is likely due to the differences between the two sites in soil type, soil structure, and water content, which was lower at the CL site (Supplementary Fig. 8) and may limit protists’ movement through the soil.

## Conclusions

Here, we present the first comprehensive characterization of protist dynamics and community assembly in the rhizosphere of plants as they undergo different phenological stages in a large-scale, multi-site field experiment. We demonstrate that environmental filtering is a dominant determinant of protist community properties. Our results also provide evidence that plant control over the physicochemical environment at the rhizosphere level, and likely the well-known regulation of root-associated bacterial communities, are critical drivers of protist community composition and dynamics. Based on these results and considering the well-known effect of rhizodeposits on bacterial and fungal communities, we hypothesize the following mechanisms for protist community assembly in the rhizosphere: As the plant enters the soil, it selects for specific bacterial communities by modifying physicochemical conditions through root exudates and other rhizodeposits. These plant-filtered microbial populations encourage protist populations that migrate toward the rhizosphere. Bacterial and fungal populations are known to change as the plant develops, and these changes may lead to a succession of protist communities in the rhizosphere. Finally, during senescence, the plant loosens its control over its microbial populations (possibly due to declines in root exudation), which translates into less complex and dense protist networks. Future studies combining datasets from different trophic levels or relying on the reconstruction of trophic complexity in the rhizosphere can help clarify the mechanisms that mediate cross-kingdom community assembly in the rhizosphere microbiome. As protists are key contributors to plant nutrient availability and bacterial community composition and abundance, mapping and understanding their patterns in rhizosphere soil is foundational to understanding the ecology of the root-microbe-soil system.

## Methods

### Sampling sites

The two sampling sites used in this study are part of a long-term experiment by the Noble Research Institute aiming to understand the factors that regulate switchgrass establishment in marginal soils. Each plot measures 22 m by 27 m. The sandy loam site (SL) is located in Burneyville, Oklahoma (33.882083, − 97.275233), and the clay loam site (CL) is located in Ardmore, Oklahoma (34.172100, − 97.07953). Soil pH, soil organic matter, water content, and plant available N and P were determined from soil samples collected from each site prior to the start of the experiment following common analytical procedures. Briefly, 10 g of soil were used for determination of gravimetric moisture, pH measurement in water, organic matter content using combustion, and plant available P using the Mehlich III extraction method [78]. Same amount of soil was used for KCl extraction and the extract used to measure NH_4_^+^ and NO_3_^−^ content using colorimetric assays. Soil properties included in this analysis are presented in the Supplementary Dataset 1 (Tab 1 and Tab 2).

### Soil sampling

Five-hundred SG seedling plants (*Panicum virgatum*) were planted in each cultivated site in May 2016, and 30 were randomly selected from each site for continuous rhizosphere and bulk soil sampling using a 5 cm diameter by 20 cm deep soil corer. These selected plants were sampled at five sampling points corresponding to different phenological stages of the switchgrass plants: T1—vegetative growth (June), T2—late vegetative growth (July), T3—reproductive growth (August/September), T4—maximal growth (October), and T5—senescence (November). Roots were separated from each core and transferred to a 50 ml centrifuge tube, while leftover soil was labeled as bulk soil and stored at − 80 °C until further processing, yielding a total of six-hundred samples. Separated roots were processed immediately to wash the 1–2 mm of attached soil (rhizosphere soil) for DNA extraction as follows: Tubes containing the roots received 50 ml of 1X phosphate buffer supplemented with 0.35% tween 20, inverted 3–4 times, vortexed for 10 s, and sonicated at a frequency of 100 (1/s). Samples were then centrifuged at 2500×*g* for 5 min. Roots were removed with sterile tweezers, and the leftover material was filtered through a sterile funnel made of a polypropylene mesh with 1 mm pores. The flow-through liquid was collected in a 50 ml centrifuge tube and centrifuged at 2500×*g* for 5 min, the supernatant removed, and the residual soil stored at − 80 °C. For uniformity purposes, aliquots of bulk soil were also washed and concentrated with the same procedures used for the rhizosphere soil, prior to DNA extraction.

### DNA extraction

Aliquots of 0.2 g of washed soil (rhizosphere or bulk soil) were transferred to a 2-ml Lysing Matrix E tube (MP Biomedicals, Solon, OH, USA), which received 500 μl of extraction buffer (5% CTAB, 0.5 M NaCl, 240 mM K_2_HPO_4_, pH 8.0) and 500 μl of 25:24:1 phenol:chloroform:isoamyl alcohol. The samples were then bead beaten in a Fast Prep instrument (MP Biomedicals, Solon, OH, USA) at 4 m/s for 30 s, and centrifuged at 16,000 g for 5 min. The supernatant was transferred to a MaXtract high density tube (Qiagen, Germantown, MD, USA) containing 500 μl of chloroform:isoamyl alcohol (24:1), and the lysis procedure repeated and supernatants collected in their corresponding tubes. The samples were centrifuged at 10,000×*g* for 1 min at 4 °C, and the supernatants transferred to a microcentrifuge tube containing 1 ml of isopropanol and 1 μl of linear acrylamide (Ambion, Grand Island, NY, USA). The DNA/RNA mixture was precipitated by incubating for 10 min at room temperature and centrifuged at 10,000×*g* for 5 min at 4 °C, and the isopropanol removed. The obtained pellet was washed with 70% ethanol and centrifuged at 10,000×*g* for 1 min at 4 °C. The ethanol was completely removed, and the pellet dissolved in DEPC-treated water. The crude extracts were transferred to a 96-well plate and purified using magnetic beads as follows: Each sample received 1.2× volume of 2% magnetic beads (Speedbeads, GE Healthcare, Chicago, IL) in 18% polyethylene glycol 8000, 1 M NaCl, 10 mM Tris-HCl, pH 8, 1 mM EDTA pH 8, 0.05% Tween 20. The plate was then incubated in a shaking incubator at 100 rpm for 10 min. The plate was placed on a magnetic stand and allowed to settle for 5 min, and the supernatant removed. Each well was washed twice with 200 μl of 80% ethanol and incubated for 1 min. Then, the ethanol was removed, the samples left to dry for 5 min, and the beads were eluted with 30 μl of elution buffer. The samples were transferred to a shaking incubator and incubated at 500 rpm for 5 min, and transferred back to the magnetic stand for 5 min. The resulting supernatant containing the DNA was transferred to a clean plate and the DNA concentration determined with the use of a Qubit fluorometer (Thermofisher Scientific, Waltham, MA). Out of the 600 samples, 582 yielded DNA that was of sufficient quality for amplicon library preparation. From the 582 samples, 293 belonged to the CL site (145 from the rhizosphere and 148 from bulk soil), and 289 from the SL site (142 from rhizosphere and 147 from bulk soil). Detailed information can be found in the Supplementary Dataset 1, Tab 4.

### Amplicon library preparation and sequencing

The amplicon libraries were prepared with a two-step barcoding approach as described by Herbold et al. [79] with some modifications. First, the target markers were PCR-amplified with diagnostic primers synthesized with a 16 bp head sequence (5′-GCTATGCGCGAGCTGC-3′, modified from Rudi et al. [80]) at the 5′ end for 25 cycles. After the 25 cycles, the PCR was paused, and a second set of primers consisting of the 16 bp head sequence and a library-specific 8 bp barcode [81] was added and amplified for 5 more cycles. Each PCR reaction (45 μl in the first step, and 50 μl in second step) consisted of 10 ng of DNA template, 1 unit of Titanium Taq DNA Polymerase (Takara Mirus Bio Inc., WI, USA), 100 ng of bovine serum albumin, 1× Titanium Taq PCR buffer, 0.2 mM dNTP mix, 0.2 μM of forward and reverse diagnostic primers, and 5 μM of the library-specific barcodes (added during the last 5 PCR cycles). Thermocycler conditions were 95 °C for 3 min; 95 °C for 30 s, 60 °C (for diagnostic primers), or 52 °C (barcodes) for 30 s; 73 °C for 5 min. Obtained PCR products were inspected by gel electrophoresis, purified using magnetic beads following the protocol for magnetic purification described in the DNA extraction section, and quantified using a Qubit fluorometer. Products were equimolarly combined and concentrated by bead purification to create sequencing libraries, which consisted of 200 samples at a time (200 out the 600 samples), yielding a total of 3 libraries. One microgram of each pooled library was used for the ligation of adapters for Illumina sequencing using the NEBNext Ultra II DNA Library Prep kit for Illumina (New Englands Biolabs). Each adapter-ligated library was quantified by qPCR using the NEBNext Library Quantification Kit. Each library was spiked with 10% phiX and sequenced on an Illumina Miseq using the Miseq Reagent kit v3.

Before amplifying all our samples, we tested two-sets of primers, targeting the V1V2 and V9, 18S rRNA regions, for the characterization of soil protist communities in our soils. The V1V2 primers were those published by Fonseca et al. [82], FO4 (5′-GCTTGTCTCAAAGATTAAGCC-3′) and R22 (5′-CCTGCTGCCTTCCTTRGA-3′). The V9 primers were previously published by Amaral et al. [83], 1380F (5′-CCCTGCCHTTTGTACACAC-3′), and 1510R (5′-CCTTCYGCAGGTTCACCTAC-3′). The results showed that, when used in our soil samples, the V1V2 amplified more non-target sequences than the V9 primers (Wilcoxon test *p* = 4.9 × 10^−7^), with 46.6% and 26.4% of the total belonging to fungi (for the V1V2 and V9 markers, respectively; Supplementary Fig 9). Our analysis also showed that the V9 primers amplified significantly more sequences (Wilcoxon test *p* = 7.3 × 10^−9^) belonging to the protist division Alveolata (27.8% for V9 vs. 4% for V1V2), and also detected sequences belonging to the division Apusozoa, Hacrobia, Protalveolata, and the phyla Mesomycetozoa and Rhodophyta which were not amplified by the V1–V3 primers. Since the V9 primers outperformed the V1V2 pair in representing protists and discriminating fungal sequences (Supplementary Fig. 9), our subsequent analyses were conducted with the V9-18S rRNA primers.

### Sequence analyses

Libraries were demultiplexed based on their unique barcodes using custom scripts and trimmed to the same length. Sequences were dereplicated and sorted by decreasing abundance using USEARCH v11 [84]. The dereplicated sequences were denoised, de-novo chimera filtered, and zero-radius OTUs (ZOTU) generated using unoise3 from USEARCH v11. Resulting ZOTUs, which are a form of amplicon sequence variants (ASVs), were screened against the NCBI nucleotide database using Blastn with an e-value of 1e-5 and keeping 100 hits. The Blast file was imported into MEGAN Community edition v.6 [85] software for taxonomic parsing to identify ZOTUs of protist origin. Filtered ZOTUs were taxonomically characterized against the PR2 database v.4.12.0 [34] using Sintax (USEARCH v11) with a cutoff of 0.8, and genus as the maximum taxonomic level. Total sequences were mapped against protist ZOTUs at a 97% identity and an abundance table was generated that was subsequently transformed into a biom table. Protists ZOTUs were then aligned using Clustalw, and the alignment was used to generate a phylogenetic tree with IQ-TREE 2 [86] using the model GTR+F+R10 (identified using model finder) and ultrafast bootstrap approximation (UFBoot) with 1000 replicates. The abundance table, mapping file, and phylogenetic tree were imported to the R software using the Phyloseq package [87].

### Data analyses

Once imported into R, 43 underperforming libraries (with less than 1000 protist sequences) were removed from the dataset. For alpha diversity, measured as Observed Richness and the Shannon Index, the libraries were subsampled to the minimum number of sequences 100 times using a seed number of 3. A rarefaction analysis was performed to show that this level of subsampling represented the communities for the chosen diversity indices (Supplementary Fig. 1). Temporal variability of within-sample diversity was assessed by calculating the coefficient of variation (CV) for observed richness and the Shannon index for each plant and its corresponding rhizosphere and bulk samples in both field sites through time [88]. Individual values were used to determine the per sample median and mean values across environments (bulk/rhizosphere) per sampling site, with higher values indicating more variables communities [88]. Statistical significance of the differences in alpha diversity and temporal variation was assessed using the Kruskal-Wallis test and pairwise comparisons with the Wilcoxon test and the Benjamini-Hochberg method for *p* value adjustment. For community composition analyses (beta-diversity), data was normalized using the variance stabilization approach in DESeq2 [89], and a weighted Unifrac distance matrix was generated using the vegan package. The obtained distance matrix was ordinated using multidimensional scaling in Phyloseq. The samples were categorized based on sampling site (SL, CL), environment (bulk, rhizosphere), sampling time (T1 to T5); the effect of these categories on data variation was tested with Adonis (nonparametric permutation multivariate analysis of variance), performed with 1000 permutations. Temporal dispersion of community composition was assessed using *betadisper* and *permutest* (R package: vegan). Correlations between environmental data and community composition were tested using *envfit* (R package: vegan). Environmental data was fitted onto the ordination space with a gam model using *ordisurf* (R package: vegan). Significant differential abundance of protists groups in the rhizosphere relative to the bulk was determined using DESeq2 (*p* < 0.01), which adjusts *p* values using false discovery rate (FDR) for multiple comparisons. For the differential abundance analysis, the data was agglomerated to the maximum identified taxonomic level for each ZOTU, and the data are discussed as differential abundance of protists populations rather than for exact sequence variants. Preferential feeding or nutrition strategies for the protists populations was described only for those protists identified at the genus level based on published reports [17, 35, 38, 90–96].

### Network construction and analysis

To investigate the dynamics of protist community patterns over time in both marginal soils, we used random matrix theory (RMT)-based co-occurrence association network analysis. Networks were constructed for rhizosphere and bulk soil at each time point based on center log ratio transformed abundance data, which was normalized using the Microbiome R package. Prior to normalization, the data was subsetted for each sampling site (SL/CL), environment (bulk/rhizosphere), and time point (T1 to T5), and underperforming samples (with less than 1000 sequences) removed while keeping a minimum of 10 replicates per dataset. Only ZOTUs detected in at least 70% of each subset of replicated samples were used for network reconstruction. Network reconstruction was conducted with the Molecular Ecological Network Analyses pipeline (MENAP, http://ieg4.rccc.ou.edu/mena/) with the following settings: for missing data fill blanks with 0.01 if data have paired values; do not take logarithm as the data was already CLR normalized; use Spearman Correlation similarity matrix; calculate by decreasing cutoff from the top; and for speed selection, regress Poisson distribution only. RMT was used to automatically identify the appropriate similarity threshold for network reconstruction [97, 98]. Modules were detected using the greedy modularity optimization method and network topological properties, including number of nodes, links, and modules, were calculated according to Deng et al. [43]. Proportion of negative over positive correlations was also calculated from the outputs of MENAP. The connectivity of each node was determined based on its within-module connectivity (*Zi*) and among module connectivity (*Pi*) [99], and used to classify the nodes based on their topological roles that they play in the network (Table 1 and Supplementary figure 2). The general classification was based on categories defined in Deng et al. [43] that considers four categories: module hubs (highly connected nodes within modules, *Zi* > 2.5), network hubs (highly connected nodes within modules, *Zi* > 2.5, *Pi* > 0.62), connectors (nodes that connect modules, *Pi* > 0.62), and peripherals (nodes connected in modules with few outside connections, *Zi* < 2.5 and *Pi* < 0.62) [43, 64, 97].

### Inferring community assembly mechanisms

The relative influences of community assembly processes were assessed by a phylogenetic bin-based null model framework, iCAMP, which was recently reported with substantially improved performance [44]. Briefly, iCAMP divided taxa into different phylogenetic groups (bins) to ensure adequate phylogenetic signal to infer selection from phylogenetic diversity; then, the processes (selection, dispersal, and drift or others) dominating each bin were identified, according to the deviation of observed phylogenetic and taxonomic diversity from random patterns simulated by null models; finally, the relative abundance of bins governed by each process was aggregated to evaluate its influence on entire community assembly. The rarefied protist ZOTU table was used to be applicable to ecological null model. Then, the analysis was performed separately for the two sites, using the “iCAMP version 1.2.9” with recommended default settings on a pipeline built on Galaxy platform (http://ieg3.rccc.ou.edu:8080). Results were summarized for each group of samples from the same habitat (rhizosphere or bulk soil) and the same time point, and then the succession of relative importance of each ecological process was compared between habitats and sites. The significance of differences was calculated based on bootstrapping with 1000 replications.

## Supplementary Information

**Additional file 1 Supplementary Figure S1**. Mean rarefaction curves for bulk and rhizosphere protist communities for different sampling times (T1 to T5) corresponding to different developmental stages of switchgrass. Rarefaction curves were determined for the Shannon index and Observed species. A) Rarefaction curves for the Sandy Loam (SL) site, B) for Clay Loam (CL) site. **Supplementary Figure S2**. Ordination plot depicting community structure of protist communities in the CL and SL sites. The trend surface of the variable pH was plotted onto the ordination space using the *ordisurf* function of the vegan package. CL= Clay Loam, SL = Sandy Loam; n = 30 for SL, n= 29 for CL. **Supplementary Figure S3**. Alpha diversity dynamics of rhizosphere and bulk soil protist communities during different sampling times. The sampling times correspond to different developmental stages of switchgrass. In each boxplot, a point represents a replicated sample per sample site and its calculated diversity index, and the diamond symbols represent the mean. The box boundaries represent the first and third quartiles of the distribution and the median is represented as the horizontal line inside each box. Boxplots whiskers span 1.5 times the interquartile range of the distribution and outliers are denoted as large points outside of the whiskers. Statistical differences were evaluated with Kruskal-Wallis test and pairwise comparisons were done using a two-sided Wilcox test with P-values adjusted using the Benjamini-Hochberg method. N values indicated at the x-axis correspond to the number of biological replicates that were used for analyses after removal of low-performing libraries (less than 1000 sequences after removal of non-protist sequences). SL = Sandy Loam site, CL = Clay Loam site. **Supplementary Figure S4** Ordination plot depicting community structure of protist communities in the bulk and rhizosphere of the CL and SL sites during the five sampling points (T1–T5). The trend surface of the variable % soil moisture was plotted onto the ordination space using the *ordisurf* function (R package: vegan). Correlations between environmental data and community composition were tested using *envfit*. CL= Clay Loam site, SL = Sandy Loam site. For the SL site n-values were as follows: Rhizosphere-T1 = 24, T2 = 21, T3 = 12, T4 = 20, T5=28; bulk-T1 = 30, T2 = 26, T3 = 29, T4 = 30, T5 = 29. For the CL site n-values were: Rhizosphere-T1 = 24, T2 = 19, T3 = 24, T4 = 15, T5 = 21; bulk-T1 = 29, T2 =29, T3 =28, T4=29, T5 =28. **Supplementary Figure S5**. Ordination plot depicting community structure of protist communities in the bulk and rhizosphere of the CL and SL sites during the five sampling points (T1–T5). The trend surface of the variable soil pH was plotted onto the ordination space using the *ordisurf* function (R package: vegan). Correlations between environmental data and community composition were tested using *envfit* (R package: vegan). CL= Clay Loam site, SL = Sandy Loam site. For the SL site n-values were as follows: Rhizosphere-T1 = 24, T2 = 21, T3 = 12, T4 = 20, T5=28; bulk-T1 = 30, T2 = 26, T3 = 29, T4 = 30, T5 = 29. For the CL site n-values were: Rhizosphere-T1 = 24, T2 = 19, T3 = 24, T4 = 15, T5 = 21; bulk-T1 = 29, T2 =29, T3 =28, T4=29, T5 =28. **Supplementary Figure S6**. Temporal variability of protist communities for observed richness and the Shannon index. Each point represents the temporal variability of protist communities in a single plant sampled for rhizosphere and soil bulk samples. The box boundaries represent the first and third quartiles of the distribution and the median is represented as the horizontal line inside each box. Boxplots whiskers span 1.5 times the interquartile range of the distribution and outliers are denoted as large points outside of the whiskers. Statistical differences were evaluated with Kruskal-Wallis test and pairwise comparisons were done using a two-sided Wilcox test with P-values adjusted using the Benjamini-Hochberg method. CL= Clay Loam site, SL = Sandy Loam site. N values indicated at the x-axis correspond to the number of biological replicates that were used for analyses after removal of low-performing libraries (less than 1000 sequences after removal of non-protist sequences). **Supplementary Figure S7.** Variability of protist community composition for bulk and rhizosphere samples collected from the marginal soil sampling sites. Beta diversity dispersion was calculated using *betadisper* (package: Vegan) and group dispersions tested with a permutation-based test of multivariate homogeneity using *permutest* (package: Vegan). The ellipses represent 1-standard deviation of the group distance to the centroid. CL= Clay Loam site, SL = Sandy Loam site. N values indicated at the x-axis correspond to the number of biological replicates that were used for analyses after removal of low-performing libraries (less than 1000 sequences after removal of non-protist sequences). **Supplementary Figure S8**. Soil moisture content from the two sampled sites along sampling points. The box boundaries represent the first and third quartiles of the distribution and the median is represented as the horizontal line inside each box. Boxplots whiskers span 1.5 times the interquartile range of the distribution and outliers are denoted as large points outside of the whiskers. Statistical differences were evaluated with Kruskal-Wallis test and pairwise comparisons were done using a two-sided Wilcox test with P-values adjusted using the Benjamini-Hochberg method. CL= Clay Loam site, SL = Sandy Loam site. N values represent the number of biological replicates used for this analysis. **Supplementary Figure S9**. Comparison of relative abundance for detected eukaryotic groups using two sets of 18S rRNA primers. The box boundaries represent the first and third quartiles of the distribution and the median is represented as the horizontal line inside each box. Boxplots whiskers span 1.5 times the interquartile range of the distribution and outliers are denoted as large points outside of the whiskers. Statistical differences were evaluated with Kruskal-Wallis test and pairwise comparisons were done using a two-sided Wilcox test with P-values adjusted using the Benjamini-Hochberg method. *Groups identified at the level of phylum. Tested samples correspond to bulk soil collected during T1 from the Clay Loam site, n = 25. **Supplementary Table S1**. Protists identified as hub taxa within the rhizosphere networks from the Sandy Loam (red box) and Clay Loam (yellow box) sites. Keystone groups are module hubs and connectors which are identified based on their within-module connectivity (*Zi* > 2.5) and among-module connectivity (*Pi* > 0.62). Their removal from their corresponding networks may cause modules and networks to disassemble. Boxes filled in red color correspond to times in which a protist was identified as hub taxa in a rhizosphere network from the Sandy Loam site while yellow filled box correspond to hub taxa at the Clay Loam site. T1 to T5 indicate the different sampling times. Feeding/nutrition preferences are indicated based on published reports (see materials and methods) for protists identified at the genus level. b = bacterivore, e = eukaryvore, o = omnivore (feeds on bacteria and protists), ph = photoautotroph, m = mixotroph, pp = plant pathogen.

**Additional file 2.** Supplementary Dataset 1. Dataset for environmental data and barcodes/categories used in this study. File in excel format containing multiple tabs. Tables for the soil series from the CL and SL sites (tab 1); soil physiochemical properties (tab 2); monthly weather conditions for each site during the sampling points (tab 3); overall sequencing metadata (barcodes, categories; tab 4), pH and % gravimetric moisture for bulk soil during sampling T1 for both sites (tab 5); metadata for samples/libraries used for comparison of primers (V1V2 vs V9, tab 6).

## Data Availability

Sequencing datasets were deposited in the NCBI Sequence Read Archive under Bioproject number PRJNA661380. R scripts are available upon request.

## Abbreviations

SG: Switchgrass
CL: Clay loam
SL: Sandy loam
ZOTU: Zero-radius OTUs
