# Supplementary material for: Protist diversity and community complexity in the rhizosphere of switchgrass are dynamic as plants develop
Source: Microbiome. 2021 Apr 28;9:96. doi: 10.1186/s40168-021-01042-9 (PMC8082632; doi:10.1186/s40168-021-01042-9)
Supplement: Supplementary file 2 — Additional file 1 Supplementary Figure S1. Mean rarefaction curves for bulk and rhizosphere protist communities for different sampling times (T1 to T5) corresponding to different developmental stages of switchgrass. Rarefaction curves were determined for the Shannon index and Observed species. A) Rarefaction curves for the Sandy Loam (SL) site, B) for Clay Loam (CL) site. Supplementary Figure S2. Ordination plot depicting community structure of protist communities in the CL and SL sites. The trend surface of the variable pH was plotted onto the ordination space using the ordisurf function of the vegan package. CL= Clay Loam, SL = Sandy Loam; n = 30 for SL, n= 29 for CL. Supplementary Figure S3. Alpha diversity dynamics of rhizosphere and bulk soil protist communities during different sampling times. The sampling times correspond to different developmental stages of switchgrass. In each boxplot, a point represents a replicated sample per sample site and its calculated diversity index, and the diamond symbols represent the mean. The box boundaries represent the first and third quartiles of the distribution and the median is represented as the horizontal line inside each box. Boxplots whiskers span 1.5 times the interquartile range of the distribution and outliers are denoted as large points outside of the whiskers. Statistical differences were evaluated with Kruskal-Wallis test and pairwise comparisons were done using a two-sided Wilcox test with P-values adjusted using the Benjamini-Hochberg method. N values indicated at the x-axis correspond to the number of biological replicates that were used for analyses after removal of low-performing libraries (less than 1000 sequences after removal of non-protist sequences). SL = Sandy Loam site, CL = Clay Loam site. Supplementary Figure S4 Ordination plot depicting community structure of protist communities in the bulk and rhizosphere of the CL and SL sites during the five sampling points (T1–T5). The trend surface of the var [file 40168_2021_1042_MOESM2_ESM.docx]

**Supplementary Figure**


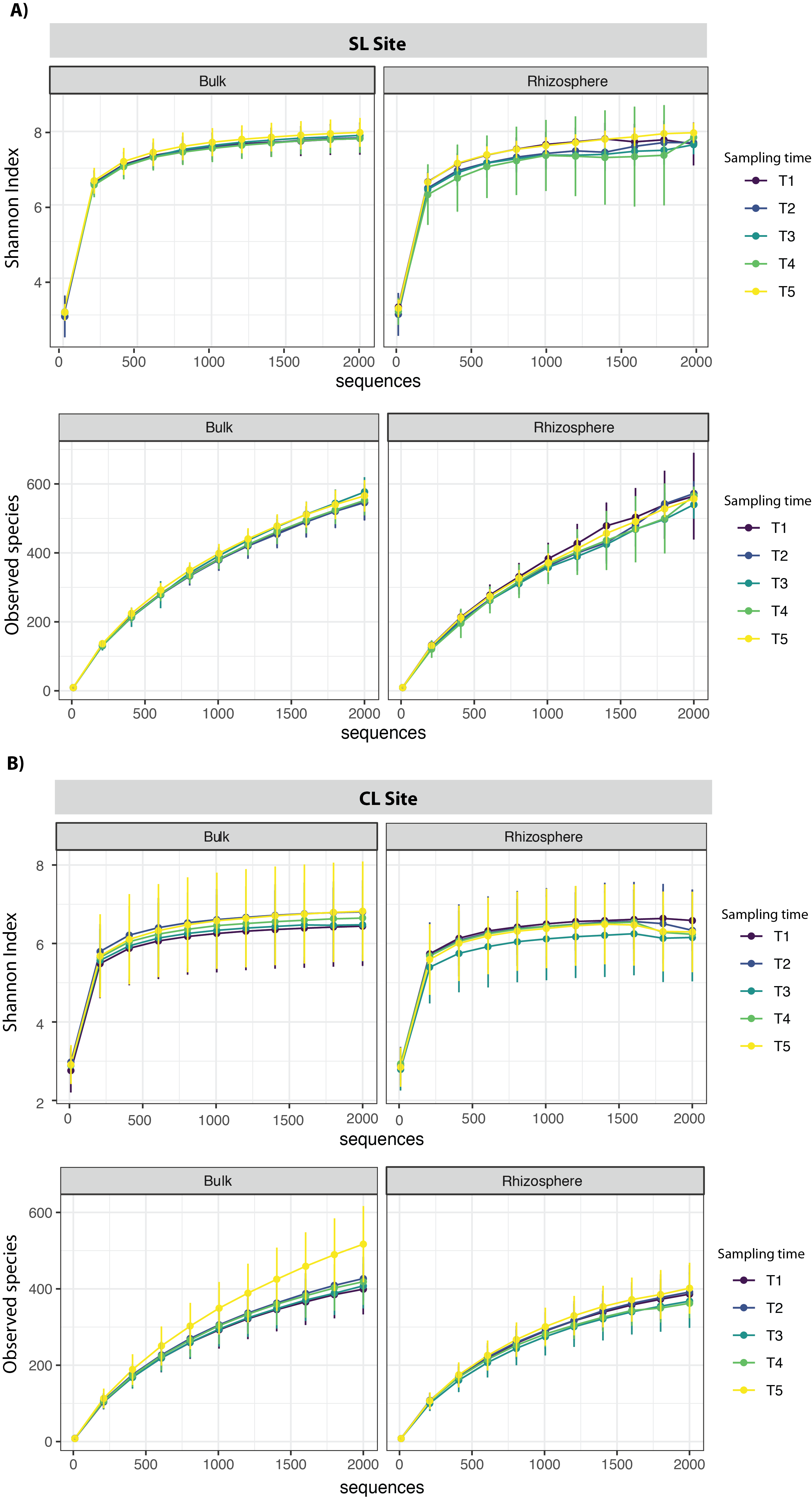


Supplementary Figure 1. Mean rarefaction curves for bulk and rhizosphere protist communities for different sampling times (T1 to T5) corresponding to different developmental stages of switchgrass. Rarefaction curves were determined for the Shannon index and Observed species. A) Rarefaction curves for the Sandy Loam (SL) site, B) for Clay Loam (CL) site.


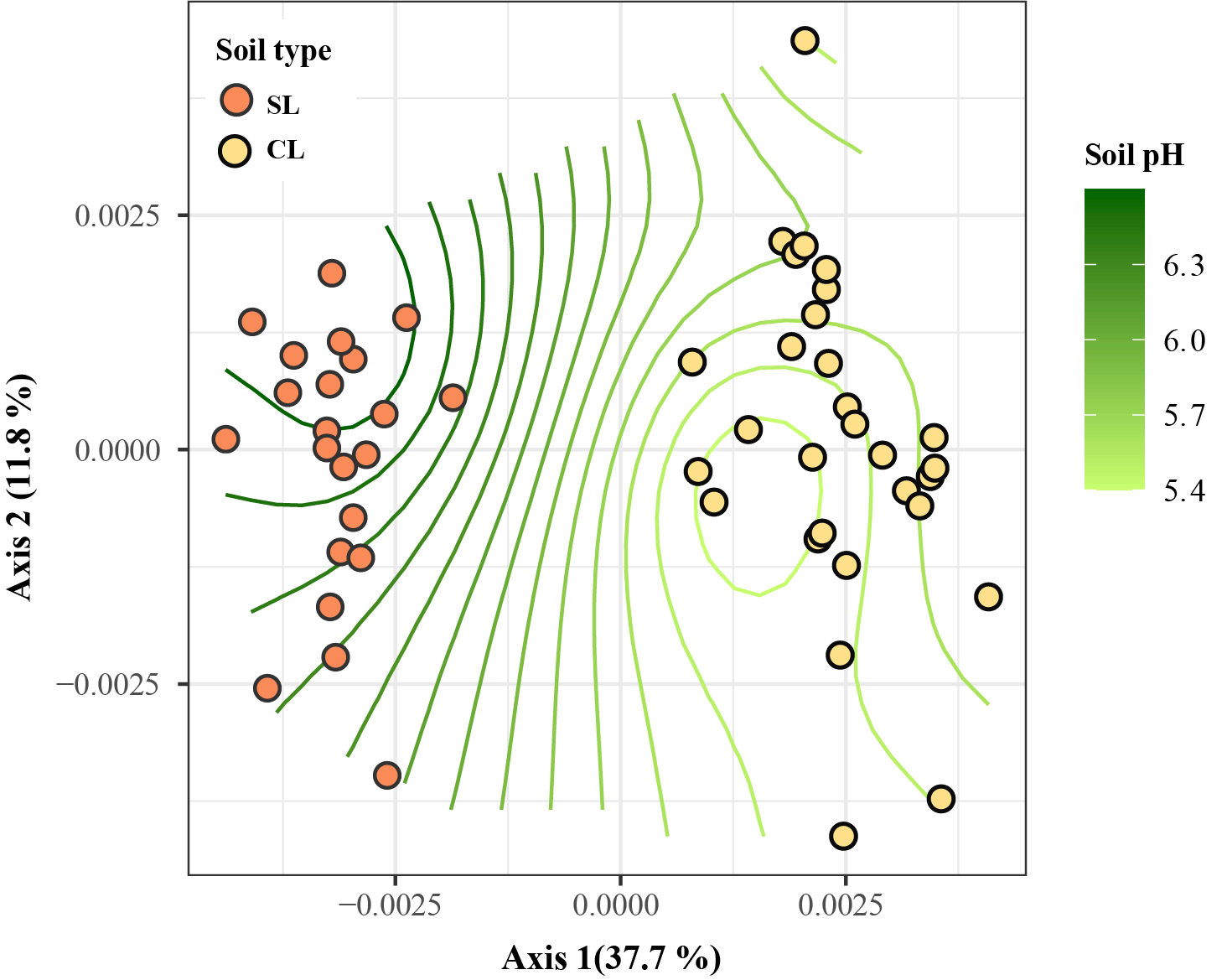


Supplementary Figure 2. Ordination plot depicting community structure of protist communities in the CL and SL sites. The trend surface of the variable pH was plotted onto the ordination space using the *ordisurf* function of the vegan package. CL= Clay Loam, SL = Sandy Loam; n = 30 for SL, n= 29 for CL.

Supplementary Figure 3. Alpha diversity dynamics of rhizosphere and bulk soil protist communities during different sampling times. The sampling times correspond to different developmental stages of switchgrass. In each boxplot, a point represents a replicated sample per sample site and its calculated diversity index, and the diamond symbols represent the mean. The box boundaries represent the first and third quartiles of the distribution and the median is represented as the horizontal line inside each box. Boxplots whiskers span 1.5 times the interquartile range of the distribution and outliers are denoted as large points outside of the whiskers. Statistical differences were evaluated with Kruskal-Wallis test and pairwise comparisons were done using a two-sided Wilcox test with P-values adjusted using the Benjamini-Hochberg method. N values indicated at the x-axis correspond to the number of biological replicates that were used for analyses after removal of low-performing libraries (less than 1000 sequences after removal of non-protist sequences). SL = Sandy Loam site, CL = Clay Loam site.


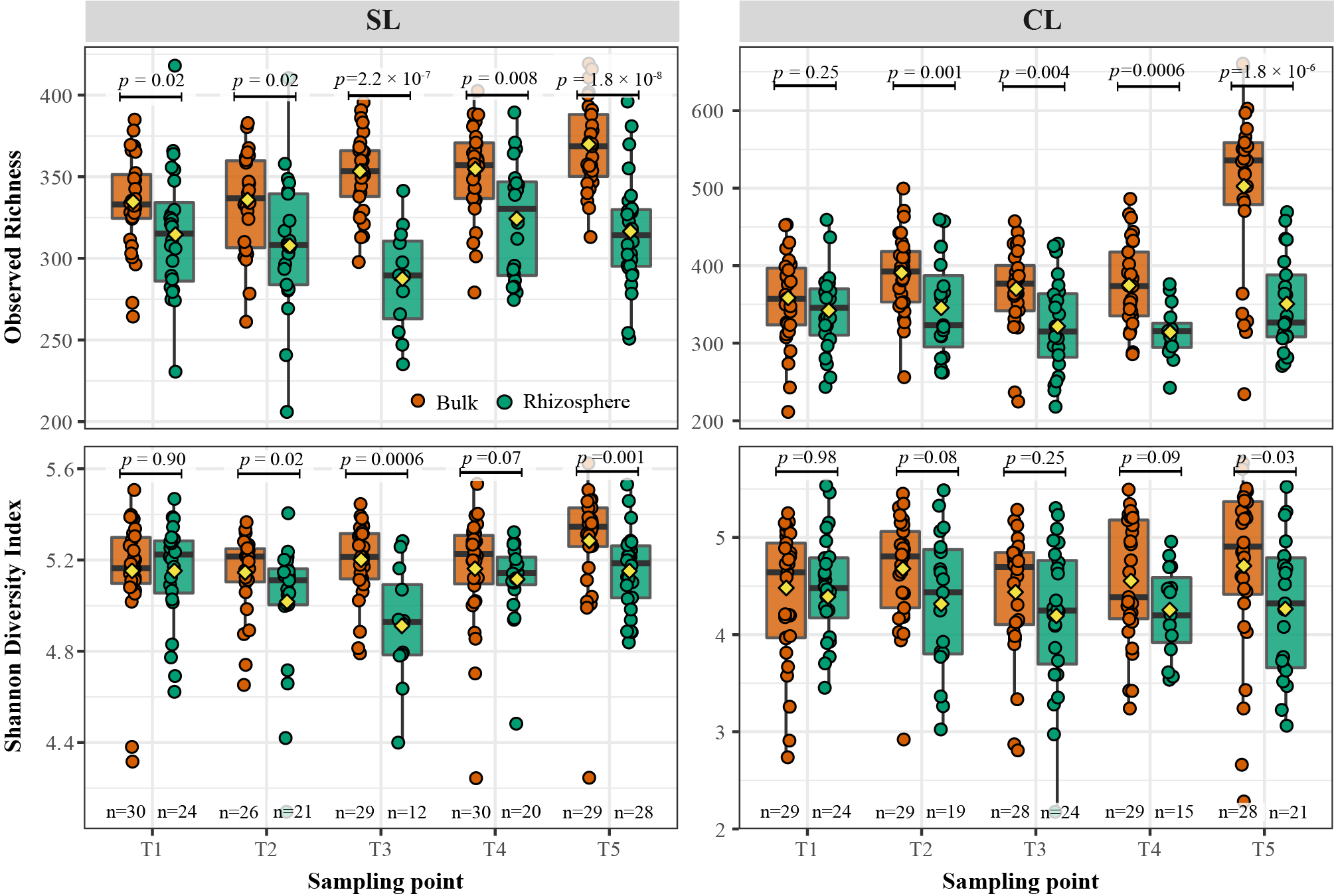


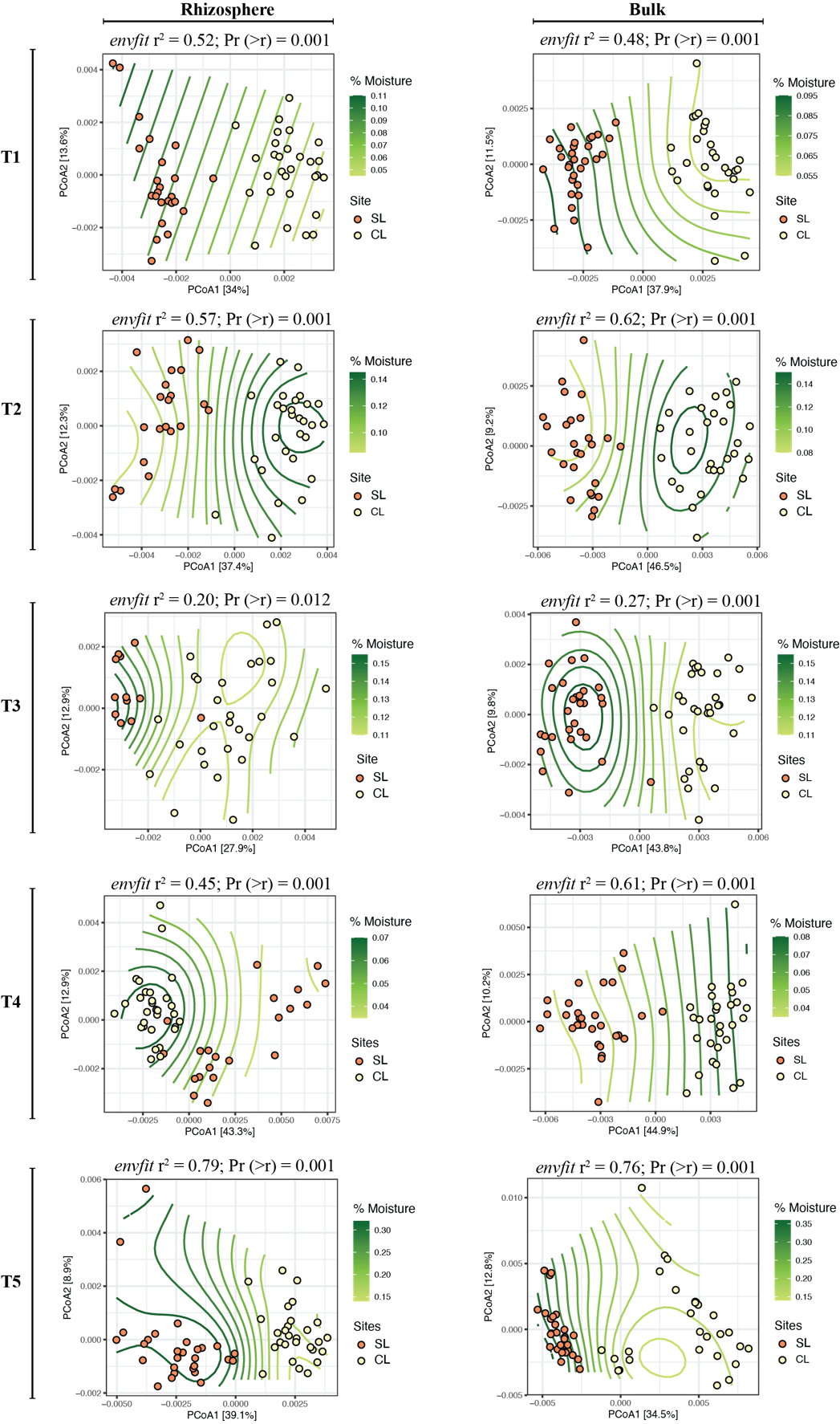


Supplementary Figure 4. Ordination plot depicting community structure of protist communities in the bulk and rhizosphere of the CL and SL sites during the five sampling points (T1–T5). The trend surface of the variable % soil moisture was plotted onto the ordination space using the *ordisurf* function (R package: vegan). Correlations between environmental data and community composition were tested using *envfit*. CL= Clay Loam site, SL = Sandy Loam site. For the SL site n-values were as follows: Rhizosphere-T2 = 21, T3 = 12, T4 = 20, T5=28; bulk-T2 = 26, T3 = 29, T4 = 30, T5 = 29. For the CL site n-values were: Rhizosphere-T2 = 19, T3 = 24, T4 = 15, T5 = 21; bulk-T2 =29, T3 =28, T4=29, T5 =28.


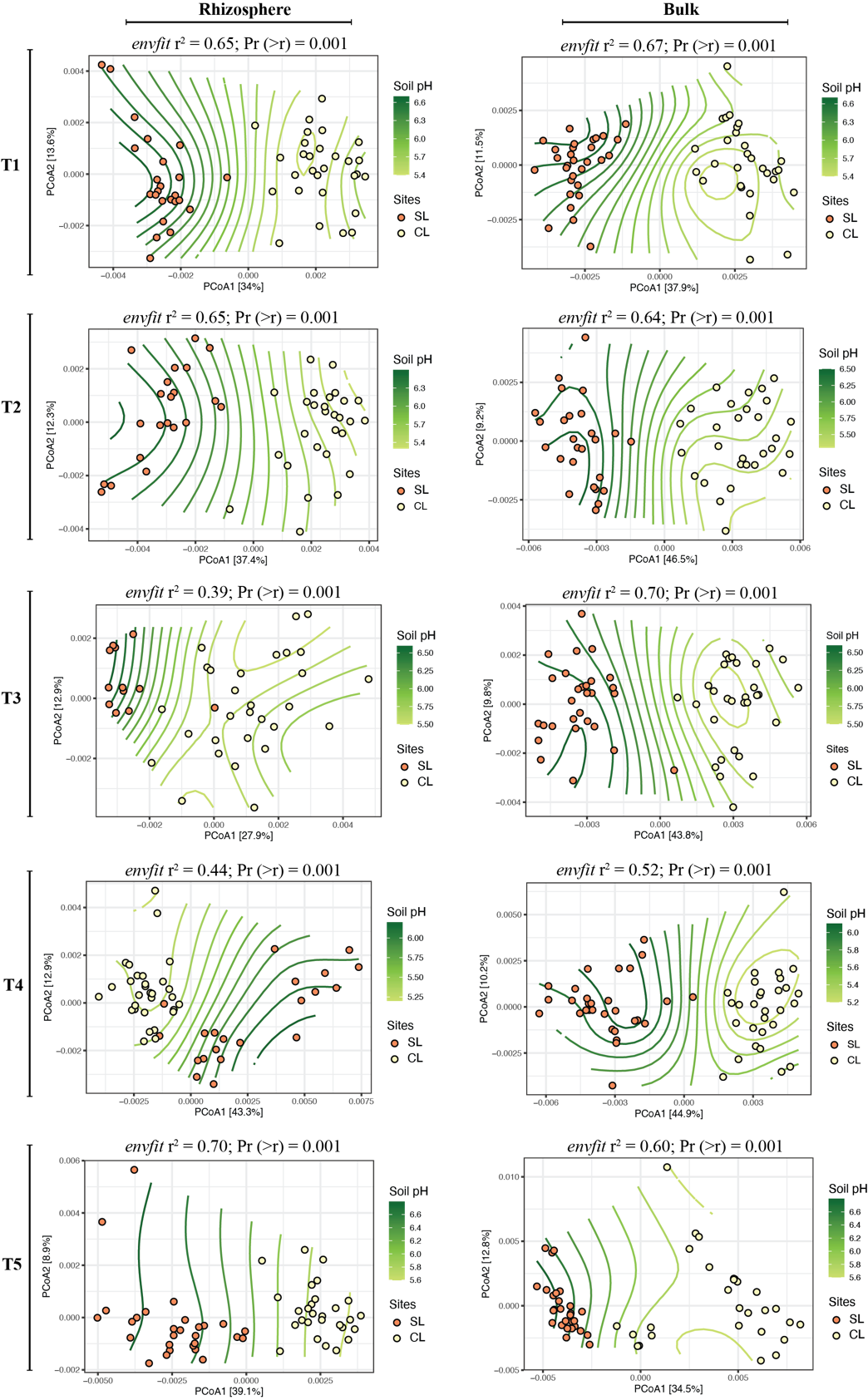


Supplementary Figure 5. Ordination plot depicting community structure of protist communities in the bulk and rhizosphere of the CL and SL sites during the five sampling points (T1–T5). The trend surface of the variable soil pH was plotted onto the ordination space using the *ordisurf* function (R package: vegan). Correlations between environmental data and community composition were tested using *envfit* (R package: vegan). CL= Clay Loam site, SL = Sandy Loam site. For the SL site n-values were as follows: Rhizosphere-T2 = 21, T3 = 12, T4 = 20, T5=28; bulk-T2 = 26, T3 = 29, T4 = 30, T5 = 29. For the CL site n-values were: Rhizosphere-T2 = 19, T3 = 24, T4 = 15, T5 = 21; bulk-T2 =29, T3 =28, T4=29, T5 =28.

Supplementary Figure 6. Temporal variability of protist communities for observed richness and the Shannon index. Each point represents the temporal variability of protist communities in a single plant sampled for rhizosphere and soil bulk samples. The box boundaries represent the first and third quartiles of the distribution and the median is represented as the horizontal line inside each box. Boxplots whiskers span 1.5 times the interquartile range of the distribution and outliers are denoted as large points outside of the whiskers. Statistical differences were evaluated with Kruskal-Wallis test and pairwise comparisons were done using a two-sided Wilcox test with P-values adjusted using the Benjamini-Hochberg method. CL= Clay Loam site, SL = Sandy Loam site. N values correspond to the number of biological replicates that were used for analyses after removal of low-performing libraries (less than 1000 sequences after removal of non-protist sequences).


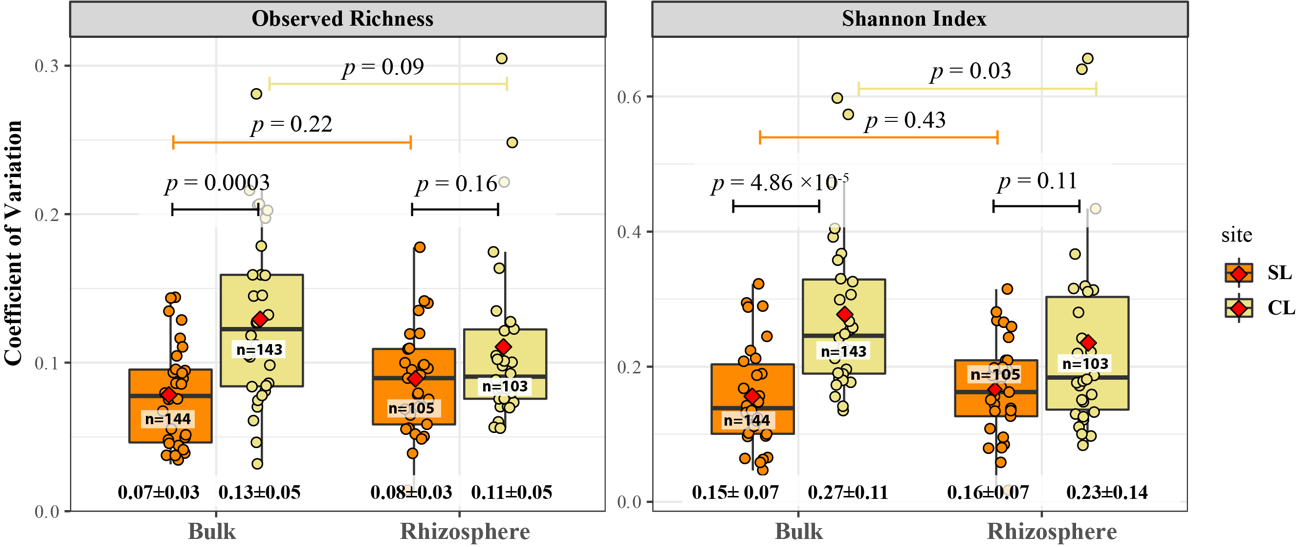

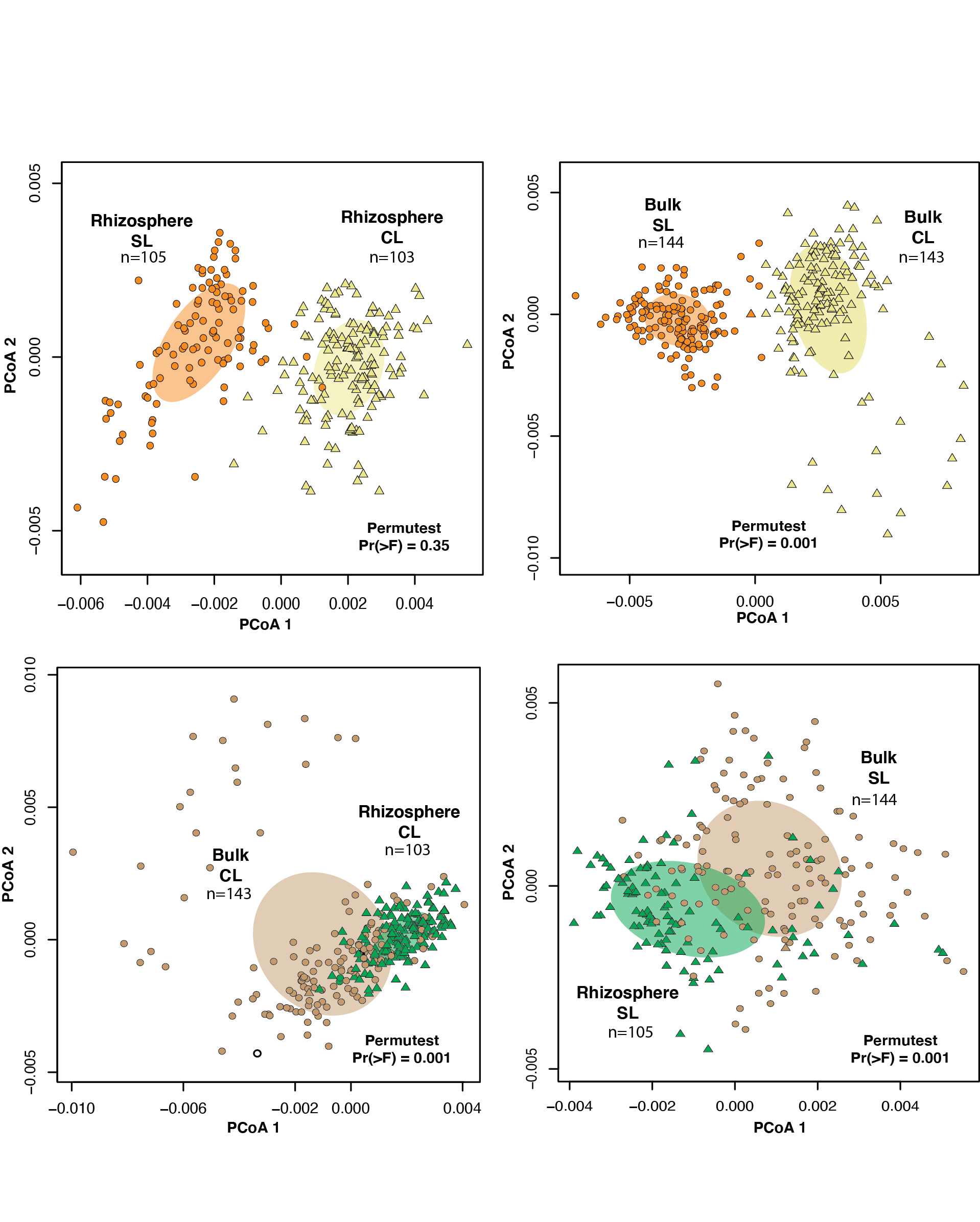


Supplementary Figure 7. Variability of protist community composition for bulk and rhizosphere samples collected from the marginal soil sampling sites. Beta diversity dispersion was calculated using *betadisper* (package: Vegan) and group dispersions tested with a permutation-based test of multivariate homogeneity using *permutest* (package: Vegan). The ellipses represent 1-standard deviation of the group distance to the centroid. CL= Clay Loam site, SL = Sandy Loam site. N values indicated in the figure correspond to the number of biological replicates that were used for analyses after removal of low-performing libraries (less than 1000 sequences after removal of non-protist sequences).


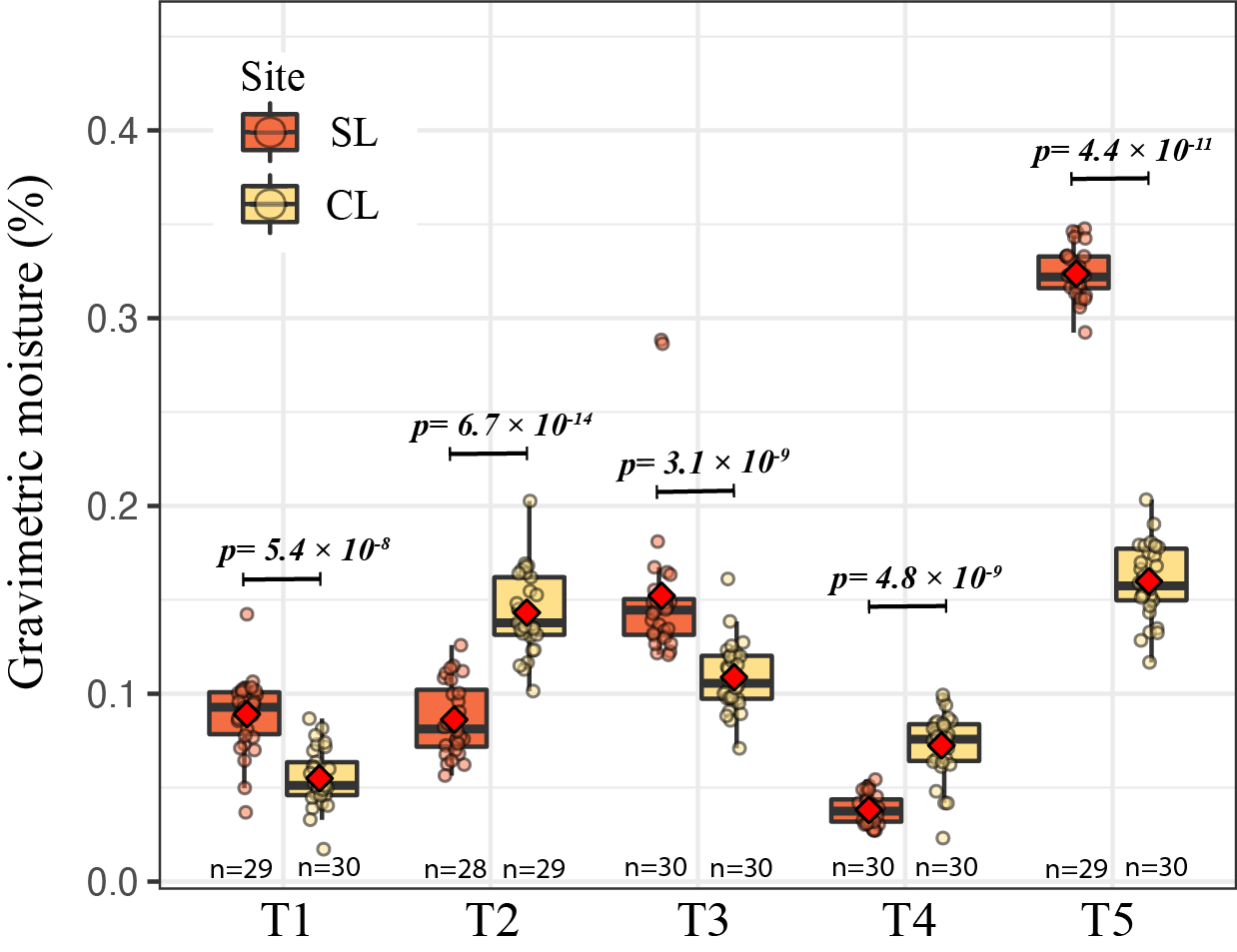


Supplementary Figure 8. Soil moisture content from the two sampled sites along sampling points. The box boundaries represent the first and third quartiles of the distribution and the median is represented as the horizontal line inside each box. Boxplots whiskers span 1.5 times the interquartile range of the distribution and outliers are denoted as large points outside of the whiskers. Statistical differences were evaluated with Kruskal-Wallis test and pairwise comparisons were done using a two-sided Wilcox test with P-values adjusted using the Benjamini-Hochberg method. CL= Clay Loam site, SL = Sandy Loam site. N values represent the number of biological replicates used for this analysis.


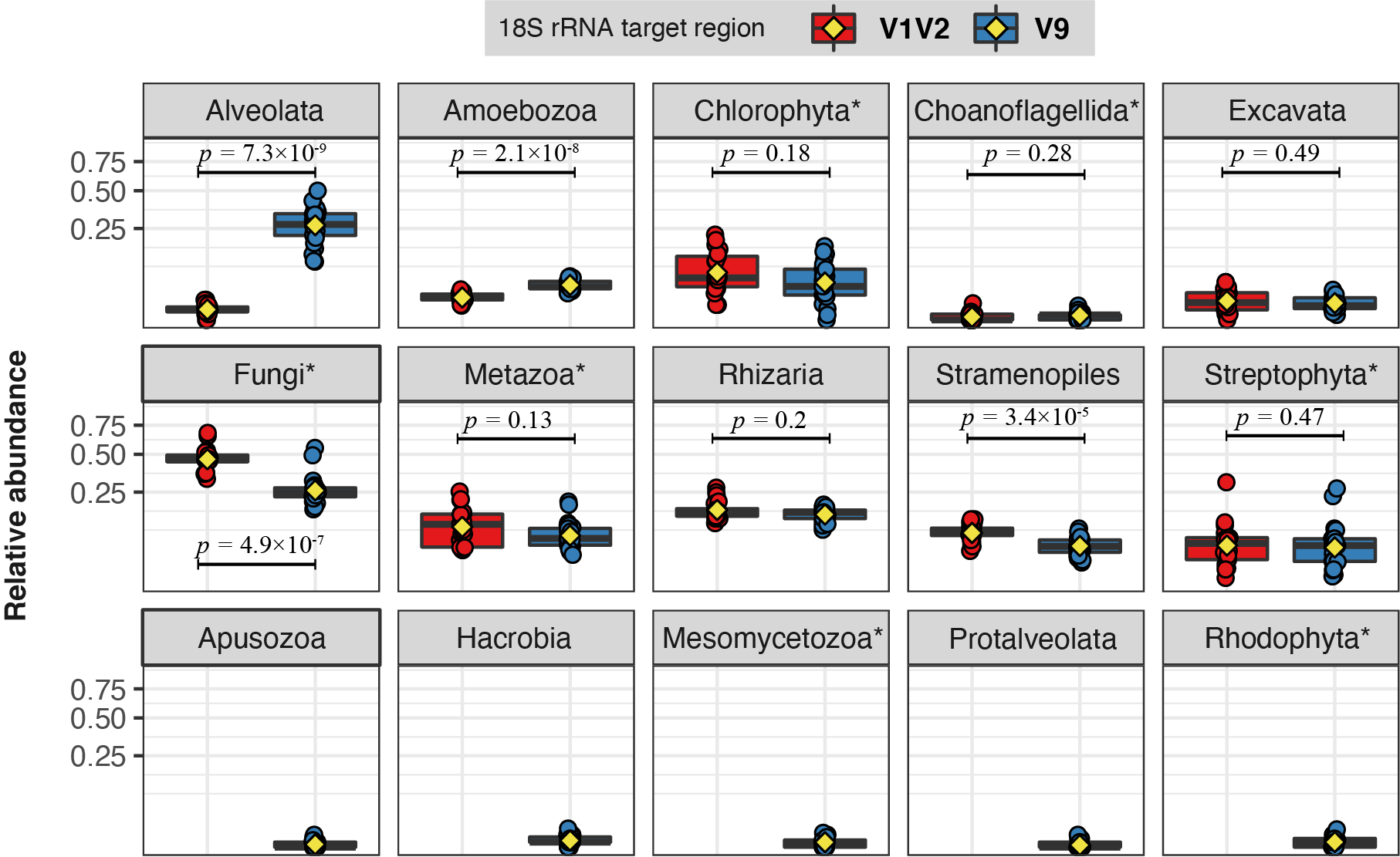


Supplementary Figure 9. Comparison of relative abundance for detected eukaryotic groups using two sets of 18S rRNA primers. The box boundaries represent the first and third quartiles of the distribution and the median is represented as the horizontal line inside each box. Boxplots whiskers span 1.5 times the interquartile range of the distribution and outliers are denoted as large points outside of the whiskers. Statistical differences were evaluated with Kruskal-Wallis test and pairwise comparisons were done using a two-sided Wilcox test with P-values adjusted using the Benjamini-Hochberg method. *Groups identified at the level of phylum. Tested samples correspond to bulk soil collected during T1 from the Clay Loam site, n = 25.

Table 1. Protists identified as putative keystone members within the rhizosphere networks from the Sandy Loam (red box) and Clay Loam (yellow box) sites.

| **Protist** | ***Zi*** | ***Pi*** | **T1** | **T2** | **T3** | **T4** | **T5** |
| --- | --- | --- | --- | --- | --- | --- | --- |
| *Pythium*^pp^ | -1.21 | 0.67 |  |  |  |  |  |
| *Bresslaua^e^* | 0.00 | 0.63 |  |  |  |  |  |
| *Paracercomonas^b^* | -0.87 | 0.63 |  |  |  |  |  |
| *Rhogostoma^o^* | -0.87 | 0.63 |  |  |  |  |  |
| *Sandonidae* | -1.06 | 0.67 |  |  |  |  |  |
| *Cercomonadida* | -0.59 | 0.63 |  |  |  |  |  |
| *Heterobolosea* | 3.06 | 0.00 |  |  |  |  |  |
| *Acanthamoeba^b^* | 2.83 | 0.00 |  |  |  |  |  |
| *Stenamoeba^b^* | -1.01 | 0.67 |  |  |  |  |  |
| *Cercomonas^o^* | 3.28 | 0.00 |  |  |  |  |  |
| *Chlorosarcinopsis^m^* | -1.07 | 0.67 |  |  |  |  |  |
| *Tetracystis^ph^* | -0.76 | 0.63 |  |  |  |  |  |
| *Thraustochytriaceae* | -0.82 | 0.72 |  |  |  |  |  |
| *Chrysophyceae* | -1.28 | 0.67 |  |  |  |  |  |
| *Cercomonadida* | 2.65 | 0.00 |  |  |  |  |  |
| *Thaumatomonas^b^* | -0.20 | 0.64 |  |  |  |  |  |
| *Cercomonadidae* | 0.14 | 0.63 |  |  |  |  |  |
| *Cryomonadidae* | -0.44 | 0.63 |  |  |  |  |  |
| *Euglypha^o^* | -0.23 | 0.63 |  |  |  |  |  |
| *Parietochloris^ph^* | 2.87 | 0.00 |  |  |  |  |  |
| *Allapsidae* | 2.58 | 0.00 |  |  |  |  |  |
| *Trebouxiophyceae* | 2.95 | 0.38 |  |  |  |  |  |
| *Polymyxa^pp^* | -1.01 | 0.67 |  |  |  |  |  |
| *Eocercomonas^o^* | 2.53 | 0.48 |  |  |  |  |  |
| *Platyophryida* | -0.83 | 0.63 |  |  |  |  |  |
| *Trinematidae* | 2.53 | 0.00 |  |  |  |  |  |
| *Acanthamoeba^b^* | -0.68 | 0.63 |  |  |  |  |  |
| *Trinema^o^* | -0.62 | 0.63 |  |  |  |  |  |
| *Paracercomonas^b^* | 0.28 | 0.67 |  |  |  |  |  |
| *Oligohymenophorea* | 2.57 | 0.24 |  |  |  |  |  |
| *Polymyxa^pp^* | 2.89 | 0.00 |  |  |  |  |  |

Keystone groups are module hubs and connectors which are identified based on their within-module connectivity (*Zi* > 2.5) and among-module connectivity (*Pi* > 0.62). Their removal from their corresponding networks may cause modules and networks to disassemble. Boxes filled in red color correspond to times in which a protist was identified as keystone element of a rhizosphere network from the Sandy Loam site while yellow filled box correspond to keystone groups at the Clay Loam site. T1 to T5 indicate the different sampling times. Feeding/nutrition preferences are indicated based on published reports (see materials and methods) for protists identified at the genus level. b = bacterivore, e = eukaryvore, o= omnivore (feeds on bacteria and protists), ph = photoautotroph, m = mixotroph, pp = plant pathogen.
